# Specific energy contributions from competing hydrogen-bonded structures in six polymorphs of phenobarbital

**DOI:** 10.1186/s13065-016-0152-5

**Published:** 2016-02-22

**Authors:** Thomas Gelbrich, Doris E. Braun, Ulrich J. Griesser

**Affiliations:** Institute of Pharmacy, University of Innsbruck, Innrain 52c, 6020 Innsbruck, Austria

## Abstract

**Background:**

In solid state structures of organic molecules, identical sets of H-bond donor and acceptor functions can result in a range of distinct H-bond connectivity modes. Specifically, competing H-bond structures (HBSs) may differ in the quantitative proportion between one-point and multiple-point H-bond connections. For an assessment of such HBSs, the effects of their internal as well as external (packing) interactions need to be taken into consideration. The semi-classical density sums (SCDS-PIXEL) method, which enables the calculation of interaction energies for molecule–molecule pairs, was used to investigate six polymorphs of phenobarbital (Pbtl) with different quantitative proportions of one-point and two-point H-bond connections.

**Results:**

The structures of polymorphs **V** and **VI** of Pbtl were determined from single crystal data. Two-point H-bond connections are inherently inflexible in their geometry and lie within a small PIXEL energy range (−45.7 to −49.7 kJ mol^−1^). One-point H-bond connections are geometrically less restricted and subsequently show large variations in their dispersion terms and total energies (−23.1 to −40.5 kJ mol^−1^). The comparison of sums of interaction energies in small clusters containing only the strongest intermolecular interactions showed an advantage for compact HBSs with multiple-point connections, whereas alternative HBSs based on one-point connections may enable more favourable overall packing interactions (i.e. **V** vs. **III**). Energy penalties associated with experimental intramolecular geometries relative to the global conformational energy minimum were calculated and used to correct total PIXEL energies. The estimated order of stabilities (based on PIXEL energies) is **III** > **I** > **II** > **VI** > **X** > **V**, with a difference of just 1.7 kJ mol^−1^ between the three most stable forms.

**Conclusions:**

For an analysis of competing HBSs, one has to consider the contributions from internal H-bond and non-H-bond interactions, from the packing of multiple HBS instances and intramolecular energy penalties. A compact HBS based on multiple-point H-bond connections should typically lead to more packing alternatives and ultimately to a larger number of viable low-energy structures than a competing one-point HBS (i.e. dimer vs. catemer). Coulombic interaction energies associated with typical short intermolecular C–H···O contact geometries are small in comparison with dispersion effects associated with the packing complementary molecular shapes.

**Graphical abstract:**
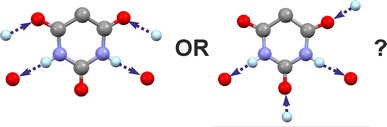
Competing H-bond motifs can differ markedly in their energy contributions

**Electronic supplementary material:**

The online version of this article (doi:10.1186/s13065-016-0152-5) contains supplementary material, which is available to authorized users.

## Background

The competition between alternative H-bonded structures (HBSs) is an important aspect of crystal polymorphism. The polymorphic forms of an organic compound may contain different HBSs which are based on the same set of (conventional [1]) H-bond donor (*D*-H) and acceptor (*A*) functions. Similarly, chemically distinct molecules with identical H-bond functions may form different HBSs, leading to the question of how molecular structure and H-bond preferences are correlated with one another.

**Fig. 1:**
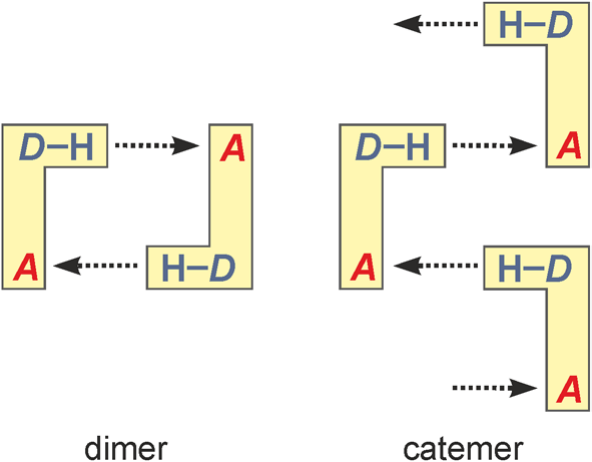
Competing H-bonded dimer (t-connection) and catemer (o-connection) structures composed of molecules with one H-bond donor (*D*-H) and one acceptor group (*A*)

The dimer versus catemer competition (Fig. 1) in small carboxylic acids [2, 3] is an example for two HBSs which are based on identical *D*-H and *A* sites but differ in the multiplicity of their H-bond connections (two-point vs. one-point). The stabilisation contribution from a molecule–molecule interaction involving two H-bonds exceeds that from each of two alternative one-point interactions significantly. Polymorphs differing in the multiplicity of their H-bond connections therefore also differ substantially in the relative distribution of energy contributions from individual molecule–molecule interactions, whereas the lattice energy differences for polymorph pairs of small organic molecules are typically very small [4–6] (<2 kJ mol^−1^ for 50 % of pairs and >7.2 kJ mol^−1^ for only 5 % of pairs [7]). This means that compensation effects arising from the packing of multiple HBS instances may be critical for the competition between one-point and multiple-point HBSs. In order to gain a better understanding of the nature of this competition, the molecule–molecule interactions in the corresponding crystals need to be examined in their entirety.

**Fig. 2:**
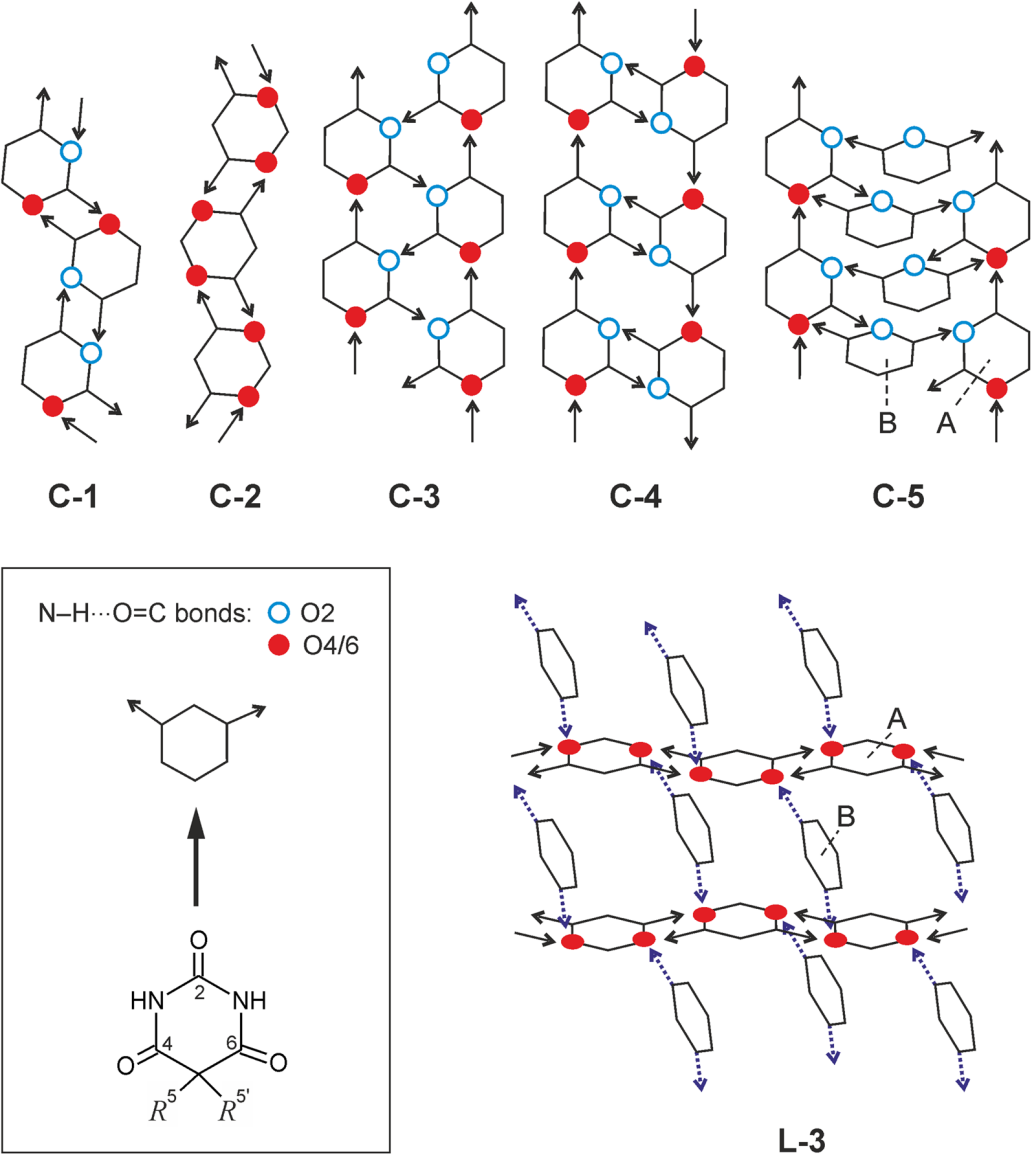
Schematic representation according to Ref. [23] of selected N–H···O=C bonded chain and layer HBSs found in derivatives of barbituric acid

Aside from small carboxylic acids [2, 3, 8] and aromatic urea dicarboxylic acids [9], competing one-point/multiple-point H-bond motifs occur for example in uracils [10], carbamazepine and its analogues [11–14], compound DB7 [15], aripiprazole [16–18], sulfonamides [19–21] and in barbiturates [22–24]. The 5,5-disubstituted derivatives of barbituric acid display a rigid 2,4,6-pyrimidinetrione skeleton whose two N–H and three carbonyl groups can serve as donor and acceptor sites, respectively, of N–H···O=C bonds. The rigid geometry of the 2,4,6-pyrimidinetrione fragment predetermines the geometries of intermolecular N–H···O=C bonds (Fig. 2) within the ensuing 1-, 2- or 3-periodic HBSs (chains, layers and frameworks). As a result of these restrictions, only a limited number of experimental HBSs are found in this set of barbiturates [23] (see Table 1), and these HBSs are based on different combinations of one-point and two-point N–H···O=C-bond connections (o- and t-connections).

**Table 1:** N–H···O=C bonded chain (**C-1** to **C-5**), layer (**L-1** to **L-6**) and framework (**F-1**, **F-2**) structures found in solid forms of barbituric acid and its 5-substituted derivatives

R^5^	R^5′^	Common name(s)	Form	Motif	CSD refcode	References
Methyl	Methyl			**C-1**	NUXTAC	[63]
Ethyl	Isopropyl	Ipral	I	**C-1**	FUFTAC	[25]
Ethyl	Butyl	Soneryl, butobarbital	RT-Form	**C-1**	ETBBAR	[64]
Ethyl	Butyl	Soneryl, butobarbital	LT-Form	**C-1**	ETBBAR01	[65]
Ethyl	Butyl	Soneryl, butobarbital		**C-1**	ETBBAR02	[66]
Allyl	Isobutyl	Sandoptal		**C-1**	FUFTIK	[25]
Ethyl	Pentan-2-yl	Pentobarbital, nembutal	I	**C-1**	FUFTEG01	[48]
Ethyl	Pentan-2-yl	Pentobarbital, nembutal	II	**C-1**	FUFTEG04	[48]
Ethyl	Pentan-2-yl or phenyl	^a^	co-crystal	**C-1**	LATMEA	[48]
Ethyl	*n*-pentyl			**C-1**	ENPBAR	[67]
Ethyl	Isopentyl	Amobarbital	II^b^	**C-1**	AMYTAL10	[68]
Ethyl	Isopentyl	Amobarbital	I^b^	**C-1**	AMYTAL11	[68]
Ethyl	But-2-enyl			**C-1**	BEBWUA	[69]
Ethyl	3-Methylbut-2-enyl			**C-1**	BECLIE	[70]
Ethyl	1,3-Dimethylbut-1-enyl			**C-1**	BEBWOU	[71]
Ethyl	1,3-Dimethylbut-2-enyl			**C-1**	JIFRIZ	[72]
Ethyl	1,3-Dimethylbutyl	α-Methylamobarbital		**C-1**	MAOBAR	[73]
Ethyl	Phenyl	Phenobarbital	CH_3_CN solvate	**C-1**	–	[35]
Ethyl	Phenyl	Phenobarbital	CH_3_NO_2_ solvate	**C-1**	–	[35]
Ethyl	1-Cyclohexen-1-yl	Phanodorm		**C-1**	ETCYBA01	[25]
Ethyl	Cyclohexyl		II	**C-1**	YOZJUU01	[49]
Allyl	Allyl	Dial		**C-1**	DALLBA	[74]
Allyl	Isopropyl	Aprobarbital	I	**C-1**	AIPBAR	[75]
F	Phenyl			**C-2**	HEKTOG	[47]
Ethyl	Ethyl	Barbital	II	**C-2**	DETBAA02	[76]
Ethyl	Pentan-2-yl	Pentobarbital, nembutal	III	**C-2**	FUFTEG02	[48]
Ethyl	Phenyl	Phenobarbital	III	**C-2**	PHBARB09	[26]
Ethyl	Phenyl	Phenobarbital	CH_2_Cl_2_ solvate	**C-2**	–	[35]
Ethyl	6-Oxocyclohexenyl	6-Oxocyclobarbital		**C-2**	OXCBAR	[77]
Cl	Cl		III	**C-3**	UXIYOQ02	[78]
Ethyl	3,3-Dimethyl-*n*-butyl	γ-Methylamobarbital		**C-3**	EMBBAR20	[79]
Ethyl	Phenyl	Phenobarbital	V	**C-3**	–	This work
Allyl	Phenyl	Alphenal		**C-3**	FUFSOP	[25]
Propenyl	1-Methylbutyl	Quinal barbitone		**C-3**	TICFER	[80]
H	H	Barbituric acid	I	**C-4**	BARBAC01	[46]
H	Ethyl		I	**C-4**	ETBARB	[81]
Methyl	Phenyl	Rutonal, heptobarbital	I	**C-4**	MPBRBL01	[25]
Methyl	Phenyl	Rutonal, heptobarbital	II	**C-4**	MPBRBL	[82]
Ethyl	Ethyl	Barbital	I	**C-4**	DETBAA01	[76]
Allyl	Cyclopent-2-en-1-yl	Cyclopal	I	**C-4**	FUFSUV	[25]
Ethyl	Butyl	Soneryl, butobarbital		**C-4** **+** **C-3**	ETBBAR03	[83]
Ethyl	Phenyl	Phenobarbital	VI	**C-5**	–	This work
Ethyl	Ethyl	Barbital	IV	**L-1**	DETBAA03	[84]
Ethyl	Pentan-2-yl	Pentobarbital, nembutal	IV	**L-1**	FUFTEG03	[48]
Ethyl	1-Methylbutenyl	Vinbarbital		**L-1**	VINBAR	[85]
Ethyl	1-Cyclohepten-1-yl	Medomin		**L-1**	CHEBAR01	[25]
H	H	Barbituric acid	II	**L-2**	BARBAC02	[46]
Ethyl	Phenyl	Phenobarbital	I	**L-3** **+** **C-2**	PHBARB07	[26]
Ethyl	Phenyl	Phenobarbital	II	**L-3** **+** **C-2**	PHBARB08	[26]
Ethyl	Cyclohexyl		I	**L-4**	YOZJUU	[49]
Isopropyl	2-Bromoallyl	Noctal	II	**L-4**	UXIYIK	[23]
Cl	Cl		I	**L-5**	UXIYOQ	[23]
Cl	Cl		II	**L-6**	UXIYOQ01	[23]
Br	Br		I	**L-6**	UXIZAD	[23]
F	F			**F-1**	HEKTIA	[47]
Br	Br		II	**F-2**	UXIZAD01	[23]

See Fig. 2 and Ref. [23] for graphical representations. R^5^ and R^5′^ are the substituents at ring position 5

^a^Co-crystal of phenobarbital and pentobarbital

^b^Nomenclature according to Ref. [25]

**Scheme 1:**
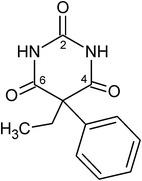
Structural formula of Pbtl

A prototypical barbiturate is phenobarbital [Pbtl, 5-ethyl-5-phenyl-2,4,6(1*H*, 3*H*, 5*H*)-pyrimidinetrione, Scheme 1] which is a sedative and anticonvulsant agent, applied as an anaesthetic and in the treatment of epilepsy and neonatal seizures. The polymorphism of Pbtl has been studied extensively [25–27] and eleven polymorphic forms, denoted by **I**–**XI**, are known [28–31]. Forms **I**–**VI** are relatively stable at ambient conditions. Their experimental order of stability at 20 °C is **I** > **II** > **III** > **IV** > **V**/**VI** [26], and they can be produced by sublimation (**I**–**VI**) or crystallisation from solution (**I**–**III**; **IV** only as an intermediate [32]) or from the melt (**IV**–**VI**). Each of the modifications **VII**–**XI** can be obtained only in a melt film preparation and only in the presence of a specific second barbiturate as a structural template (“isomorphic seeding”) [25]. Crystal structure reports exist for **I**–**III** (Table 2) [26, 33, 34], several solvates [35] and a monohydrate [36] of Pbtl.

**Table 2:** Descriptors for HBS types found in barbiturates: short HBS symbol [19] and number of o- and t-connections [*N*_o_, *N*_t_]

Type	Short HBS symbol	[*N*_o_, *N*_t_]	[*N*_o_, *N*_t_]_A_ [*N*_o_, *N*_t_]_B_ …	Pbtl form(s)
**C-1**	C4_2_[0]	[0, 2]		**X**
**C-2**	C4_2_[0]	[0, 2]		**I**, **II**, **III**
**C-3**	C4_4_[3^3^.4^2^.5]	[4, 0]		**V**
**C-4**	C4_3_[4^2^.6]	[2, 1]		
**C-5**	C5_4_.3_2_[(5^3^.6^2^.7)(5)]	[2, 1]	[3, 1][1, 1]	**VI**
**L-1**	L4_3_[6^3^-**hcb**]	[2, 1]		
**L-2**	L4_4_[4^4^.6^2^-**sql**]	[4, 0]		
**L-3**	L6_4_.2_2_[(6^4^.8.10)(6)]	[2, 1]	[2, 2][2, 0]	**I**, **II**
**L-4**	L4_3_[6^3^-**hcb**]	[2, 1]		
**L-5**	L4_3_[6^3^-**hcb**]	[2, 1]		
**L-6**	L3_2_.5_4_.4_3_[(10)(6^3^.10^3^)(6^3^)]	[2, 1]	[1, 1][3, 1][2, 1]	
**F-1**	F4_4_[6^6^-**dia**]	[4, 0]		
**F-2**	F4_3_[10^3^-**ths**]	[2, 1]		

For graphical representations, see Fig. 2 and Ref. [23]

Herein we report single crystal structure determinations for forms **IV** and **V**. A structure model for polymorph **X** was derived from an isostructural co-crystal. The polymorphs **I**–**V** and **X** contain five distinct N–H···O=C-bond motifs (or combinations of such motifs) with different quantitative proportions of o- and t-connections. Interaction energies associated with these HBSs were systematically compared using specific energy contributions of molecule–molecule interactions obtained from semi-classical density sums (SCDS-PIXEL) calculations [37–40]. An optimisation of molecular geometry was carried out and the intramolecular energy penalties of the experimental molecular geometries were determined. Using the *XPac* method [41], the new crystal data for **V**, **VI** and **X** were compared to theoretical Pbtl structures from a previous study [42].

## Results

### Hydrogen-bonded structures

The Cambridge Structural Database (version 5.35) [43] and recent literature contain the 53 unique crystal structures of barbituric acid and its 5-substituted derivatives listed in Table 1. These crystals have in common that each of the two N–H groups per molecule is engaged in a single intermolecular N–H···O=C interaction. The availability of three carbonyl groups per molecule enables various H-bond connectivity modes, whereas the inflexible arrangement of the *D* and *A* functionalities within the 2,4,6(1*H*,3*H*)-pyrimidinetrione unit predetermines the geometry of the resulting H-bonded structures. Altogether, 13 distinct H-bonded chain, layer or framework structures have been identified so far (Table 2), with one-dimensional structures, specifically the loop chains **C-1** and **C-2**, dominating this set of barbiturates (Table 1). For the purpose of classification, one has to distinguish between the carbonyl group at C2 on the one hand and the two topologically equivalent carbonyl groups at C4 and C6 on the other (Fig. 2).^1^ The observed HBSs contain different quantitative proportions of o- and t-connections, but as each NH donor function is employed exactly once, the condition

1 $$N_{\text{o}} + 2N_{\text{t}} = 4$$

applies throughout, where *N*_o_ and *N*_t_ is the number of o- and t-connections, respectively. Each [*N*_o_, *N*_t_] combination of [0, 2], [4, 0] and [2, 1] is permitted for uninodal nets. The structures **C-5** (form **VI**) and **L-3** (forms **I** and **II**) are both binodal, i.e. they feature two sets of topologically distinct molecules, whereas the layer **L-6** [23] contains three molecule types with distinct H-bond connectivity modes. In these cases, condition (1) applies for *N*_o_ and *N*_t_ parameters averaged over the HBS (Table 2).

Molecules forming the loop chains **C-1** and **C-2** (Fig. 2) are linked by two antiparallel t-connections so that [*N*_o_, *N*_t_] = [0, 2]. The underlying topology of each of **C-1** and **C-2** is that of a simple chain. In an alternative graph-set description according to Etter [44, 45], their “loops” represent ${\text{R}}_{2}^{2} \left( 8 \right)$ rings. The **C-1** type (form **X**) contains two topologically distinct ${\text{R}}_{2}^{2} \left( 8 \right)$ rings in which either two O2 or two O4/6 sites are employed, whereas in a **C-2** chain (forms **I**, **II** and **III**) only O4/6 acceptor sites are employed, and all its ${\text{R}}_{2}^{2} \left( 8 \right)$ rings are topologically equivalent.

The molecules in a **C-3** tape (form **V**) possess four o-connections so that [*N*_o_, *N*_t_] = [4, 0] (Fig. 2). Via C4/6 carbonyl groups, they form two parallel N–H···O=C bonded strands which are offset against one another by one half of a period along the translation vector. N–H···O=C bonding between the strands via C2 carbonyl groups results in fused ${\text{R}}_{3}^{3} \left( {12} \right)$ rings. Four o-connections per molecule are also present in the layer structure **L-2** [46] which has the topology of the (4,4) net and in the **dia** framework **F-1** [47].

In an **L-3** layer (forms **I** and **II**), molecules of type A are linked into **C-2** chains and B-type molecules serve as N–H···O=C bonded bridges between these chains (Fig. 2). In molecule A, the H-bond acceptor functions of the carbonyl groups at C4 and C6 are each employed twice, whereas none of the carbonyl groups of molecule B is involved in hydrogen bonding. Each molecule A forms two t-connections to A molecules and o-connections to two B molecules. There are no H-bonds between B molecules. The [*N*_o_, *N*_t_] parameters for molecules A and B are [2, 2] and [2, 0], respectively, and the overall [*N*_o_, *N*_t_] parameter combination for the **L-3** layer is [2, 1].

**Fig. 3:**
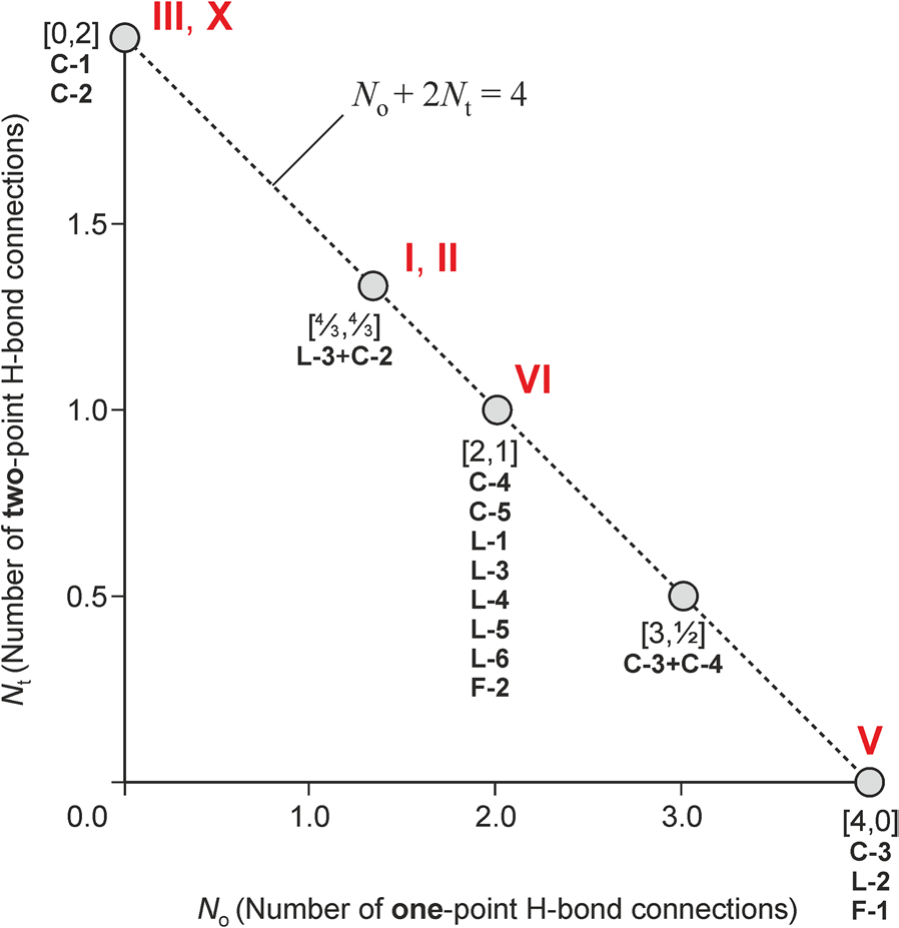
The parameters [*N*_o_, *N*_t_] for the HBS types formed by barbiturates and for two combinations of HBS types (**L-3** + **C-2** and **C-3** + **C-4**). Roman numerals indicate the relevant data points for Pbtl polymorphs

The binodal tape **C-5** (Fig. 2) is a novel structure found exclusively in the Pbtl polymorph **VI**. Molecules of type A are linked, by o-connections via C4 carbonyl groups, into two parallel strands. Additionally, the C4 and C2 carbonyl groups of molecules A and B, respectively, are employed in an asymmetrical and antiparallel t-connection. Molecule A forms also an o-connection to a second B molecule via its C2 carbonyl group. There are no H-bonds between B molecules, which serve as H-bridges between two strands. The molecule types A and B have the parameters [*N*_o_, *N*_t_]_A_ = [3, 1] and [*N*_o_, *N*_t_]_B_ = [1, 1] and the overall [*N*_o_, *N*_t_] combination for the **C-5** tape is [2, 1]. Five uninodal HBSs with [*N*_o_, *N*_t_] = [2, 1] are known, namely the **C-4** ladder, three distinct layer structures (**L-1**, **L-4**, **L-5**), each having the topology of the (6,3) net, and the **ths** framework **F-2** [23]. The connectivity and topology characteristics of the barbiturate HBSs are listed in Table 2 and an illustration of the variations in *N*_o_ and *N*_t_ is given in Fig. 3.

### SCDS-PIXEL calculations

Total PIXEL energies of individual molecule–molecule interactions (*E*_T_) can be divided into contributions from Coulombic (*E*_C_), polarisation (*E*_P_), dispersion (*E*_D_) and repulsion (*E*_R_) terms. The polarisation energy is not pairwise additive (many-body effect) so that the total PIXEL energy for the crystal, *E*_T,Cry_, differs slightly from the sum of all individual PIXEL interaction energies *E*_T,Σ_. For the Pbtl polymorphs, this difference is 2–3 kJ mol^−1^ (<2.5 % of *E*_T,Cry_; see Table 3).

**Table 3:** Crystal data and PIXEL energies of polymorphs of Pbtl

Form	**I**	**II**	**III**	**V** ^a^	**VI**	**X** ^b^
References	[26]	[26]	[26]	This work	This work	[25, 48]
CCDC refcode	PHBARB07	PHBARB08	PHBARB09	–	–	LATMEA
Space group	*P*2_1_/*n*	*P* $\overline{1}$	*P*2_1_/*c*	*P*2_1_/*n*	*P*2_1_/*n*	*C*2/*c*
*Z*′	3	3	1	2	2	1
*a* (Å)	10.70	10.74	9.55	12.76	14.67	12.67
*b* (Å)	47.26	23.40	11.85	6.76	6.90	20.69
*c* (Å)	6.80	6.72	10.81	26.85	23.03	10.25
α (°)	90	91.0	90	90	90	90
β (°)	94.2	94.5	111.6	98.8	94.1	118.5
γ (°)	90	88.4	90	90	90	90
*T*_exp_ (K)	298	173	298	173	173	173
*D* (g cm^−3^)	1.349	1.376	1.357	1.348	1.327	^d^
HBS	**C-2** + **L-3**	**C-2** + **L-3**	**C-2**	**C-3**	**C-5**	**C-1**
[*N*_o_, *N*_t_]	[4/3, 4/3]	[4/3, 4/3]	[0, 2]	[4, 0]	[2, 1]	[0, 2]
m.p. (°C) [26]	176	174	168	160	156	126
*E*_T,Cry_/Δ*E*_intra_ (kJ mol^−1^)	^c^/7.3	^c^/7.5	−118.3/3.9	−122.4/13.1	−114.9/3.7	−118.3/8.0
*E*_T,Σ_ (kJ mol^−1^)	−123.3	−122.4	−120.5	−124.1	−117.9	−121.1
*E*_T,Σ(A)_/Δ*E*_intra_ (kJ mol^−1^)	−143.1/8.9	−141.4/8.7	–	−120.9/8.5	−128.3/0.3	–
*E*_T,Σ(B)_/Δ*E*_intra_ (kJ mol^−1^)	−103.8/6.9	−104.0/8.2	–	−127.4/17.6	−107.5/7.1	–
*E*_T,Σ(C)_/Δ*E*_intra_ (kJ mol^−1^)	−122.9/6.0	−121.9/5.5	–	–	–	–
Density order	3rd	1st	2nd	4th	5th	^d^
Stability order (RT) [26]	1st	2nd	3rd	4/5th	4/5th	^e^
Stability order (calc.)^f^	2nd	3rd	1st	6th	4th	5th

^a^The matrix ($100 00\overline{1} \overline{1} 0\overline{1}$) transforms the room temperature data reported by Williams [36] (*a* = 12.66, *b* = 6.75, *c* = 27.69 Å; β = 106.9°; *P*2_1_/*c*) into a unit cell (*a′* = 12.66, *b′* = 6.75, *c′* = 26.89 Å; β’ = 99.9°*; P*2_1_/*n*) which matches our data

^b^The structure model for form **X** (Additional file 1: Section 8) was derived from the isostructural co-crystal of Pbtl with pentobarbital (the quoted CCDC refcode, unit cell data and *T*_exp_ all refer to the co-crystal)

^c^ *E*_T,Cry_ not determined because of Z*′* > 2

^d^Not applicable

^e^Exists only in a melt-film preparation and in the presence of a structurally analogous second barbiturate

^f^Based on the results of SCDS-PIXEL calculations, corrected for Δ*E*_intra_

Various aspects of the PIXEL calculation for each polymorph will be visualised in a special kind of diagram whose data points represent molecule–molecule interactions energies accounting for at least 95 % of *E*_T,Cry_, with internal HBS interactions separated from contacts between different instances of the HBS (labelled **@1**, **@2**,…). Moreover, sums of PIXEL energies will be compared in order to assess relative contributions from certain groups of interactions. The molecule–molecule interactions in each crystal structure will be ranked in descending order of their stability contributions (#1, #2, #3…), with symmetry equivalence indicated by a prime (e.g. #1/1′).

Polymorphs containing exclusively or predominantly t-connections, i.e. **X** (**C-1**), **III** (**C-2**), **I** and **II** (**C-2** + **L-3**), will be discussed first, followed by forms **V** (**C-3**) and **VI** (**C-5**). PIXEL energies do not account for differences in molecular conformation, and this topic will be discussed in a separate section. Detailed results of SCDS-PIXEL calculations are given in Additional file 1: Fig. S7 and Tables S1–S12.

### HBS type **C-1**: polymorph **X**

The structure of polymorph **X** has not been determined from single crystal data. Melt film experiments [25] indicated it to be isostructural with the co-crystal of Pbtl with 5-ethyl-5-(pentan-2-yl)barbituric acid (pentobarbital). The asymmetric unit of this co-crystal (space group *C*2/*c*) consists of a single barbiturate molecule whose R^5′^ substituent is disordered between the pentan-2-yl and phenyl groups of the two chemical components [48]. An approximate structure model for polymorph **X** was derived by removing the pentan-2-yl disorder fragment from the co-crystal structure (Additional file 1: Section 8).

**Fig. 4:**
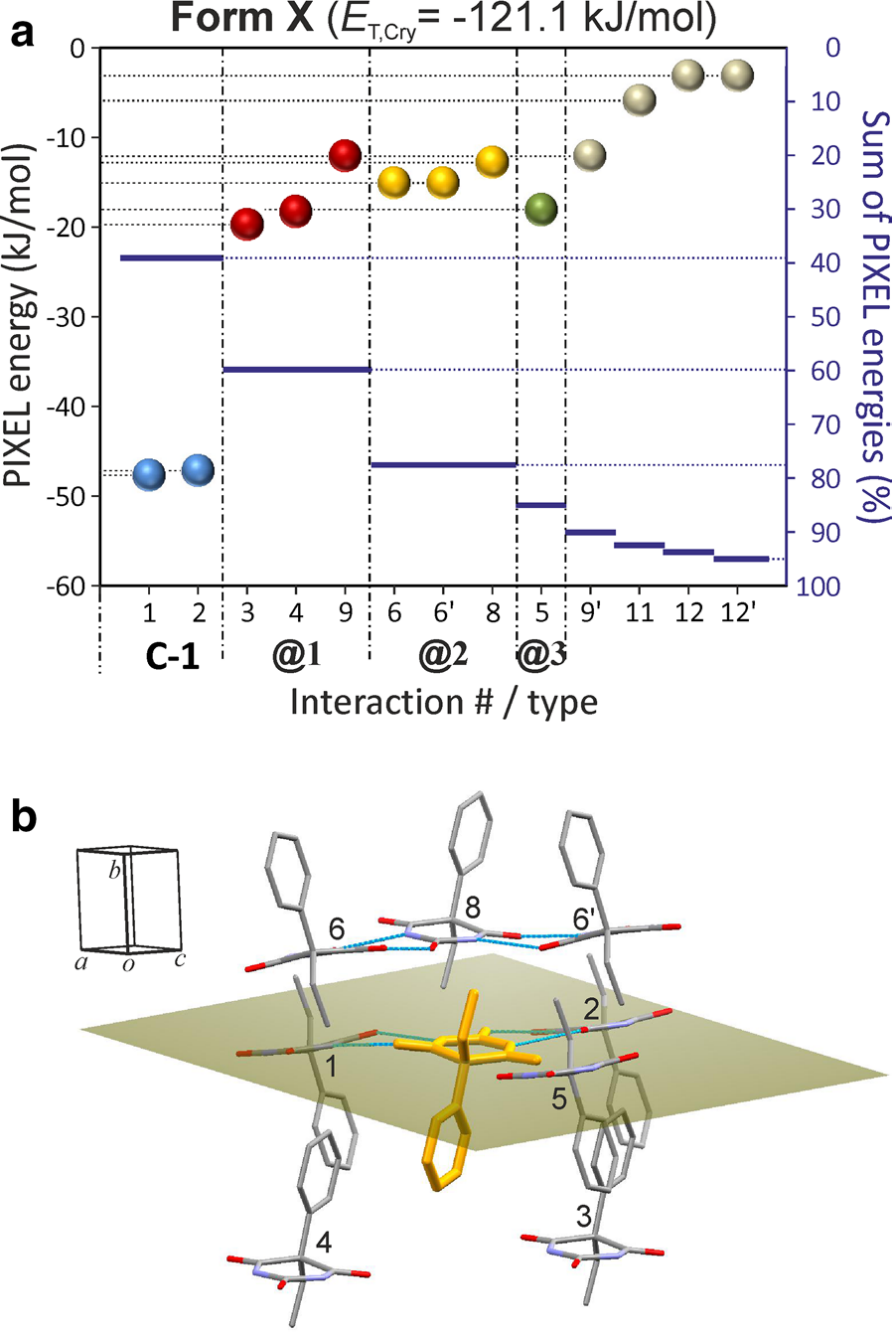
Results of SCDS-PIXEL calculations for polymorph **X**. **a** Interaction energies, represented by *balls*, are separated into internal **C-1** interactions (*blue*) and chain–chain contacts (*highlighted* **@1**, *red*; **@2**, *orange*; **@3**, *green*). The *horizontal bars* indicate cumulative PIXEL energies (summation from left to right) relative to *E*_T,Cr*y*_ (scale on the *right-hand side*). **b** The eight most important pairwise interactions involving a central molecule (*orange*). The mean plane of the pyrimidine ring of the central molecule is drawn, H atoms are omitted for clarity and H-bonds are indicated by *blue lines*

The **C-1** structure (Fig. 2) is defined by two independent t-connections with very similar interaction energies (#1: −47.5 kJ mol^−1^; *A*: O4) and (#2: −47.2 kJ mol^−1^; *A*: O2), with a crystallographic two-fold axis passing through the centre of the respective ${\text{R}}_{2}^{2} \left( 8 \right)$ ring. As expected, these interactions are dominated by the *E*_C_ term and the **C-1** tape contains no significant non-H-bonded interactions (Fig. 4a).

**Fig. 5:**
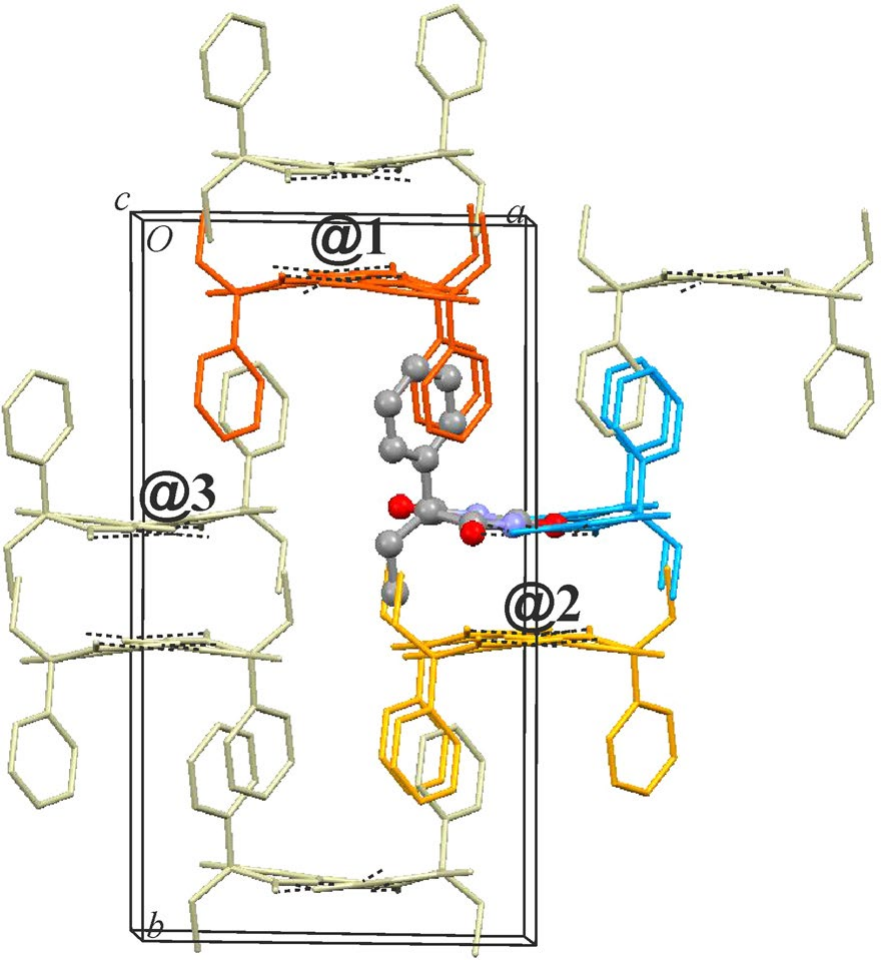
Packing diagram of polymorph **X**, showing interactions of a selected Pbtl molecule (drawn in ball-and-sticks-style) within the same **C-1** chain (*blue*) and with molecules belonging to three neighbouring chains (**@1**–**@3**; see Fig. 4). Together, hydrogen bonding and the …**@1** **@2** **@1** **@2**… stacking of chain pairs account for 78 % of *E*_T,Cry_

Each Pbtl molecule interacts with eight other molecules belonging to four different **C-1** chains, i.e. **@1** (#3, #4, #9), **@2** (#6/6′, #8), **@3** (#5) and **@4** (#9). Each of the eight interactions (PIXEL energies −19.7 to −12.1 kJ mol^−1^) is dominated by the *E*_D_ term (Additional file 1: Table S12). The chain–chain contact **@1** involves the mutual interdigitation of phenyl groups (#3, #4) and contact **@2** the interdigitation of ethyl groups (#6/6′) (Figs. 4b, 5). Internal **C-1** interactions contribute 39 % to the *E*_T,Cry_ value of −121.1 kJ mol^−1^, whilst **@1** and **@2** account for 21 and 18 %, respectively, of *E*_T,Cry_. A number of 2D and 3D packing relationships between barbiturates are based on the packing motif of the centrosymmetric chain pair **@2** [25, 49].

Each of the molecule–molecule interactions #3, #5 and #8 involves a pair of symmetry-related C–H···O contacts (H···O = 2.51–2.68 Å and CHO = 140°–170° and a significant *E*_C_ contribution (−9.1 to −9.8 kJ mol^−1^), which is however still considerably lower than the respective *E*_D_ contribution (−15.1 to −21.4 kJ mol^−1^). These C–H···O contacts are formed between the phenyl group (#3) or the CH_2_ group (#5) and the C4/6 carbonyl group not involved in classical H-bonds or between the methyl and the C2 carbonyl group (#8; for details, see Additional file 1: Table S12).

### HBS type **C-2**: polymorph **III**

The structure of **III** (space group *P*2_1_/*c*) contains one independent molecule. Its **C-2** chain (Fig. 2) possesses 2_1_ symmetry. The interaction energy of its t-connections (#1/1′) of −45.4 kJ mol^−1^ is similar to the corresponding values in **X**. The energies of the next four strongest interactions (#3, #4, #5/5′) lie between −22.1 and −19.7 kJ mol^−1^ and each of them is dominated by the *E*_D_ term (Additional file 1: Table S7). They result mainly from the pairwise antiparallel alignment of ethyl-C5-phenyl fragments in the case of #3 and from the pairwise stacking of ethyl groups with phenyl groups in the case of #5/5′. The relatively large *E*_C_ term (−13.2 kJ mol^−1^) for interaction #4 coincides with the presence of two symmetry-related (phenyl)C–H···O=C contacts (H···O = 2.53 Å, CHO = 139°) involving the C2 carbonyl group, which is not engaged in classical hydrogen bonding. However, the stabilisation contribution from *E*_D_ (−17.3 kJ mol^−1^) is still higher than *E*_C_ for interaction #4. A similar (phenyl)C–H···O=C contact geometry (H···O 2.61 Å, CHO = 151°), also involving the C2 carbonyl group, is associated with interaction #10/10′, but here the *E*_C_ contribution is just −5.5 kJ mol^−1^.

**Fig. 6:**
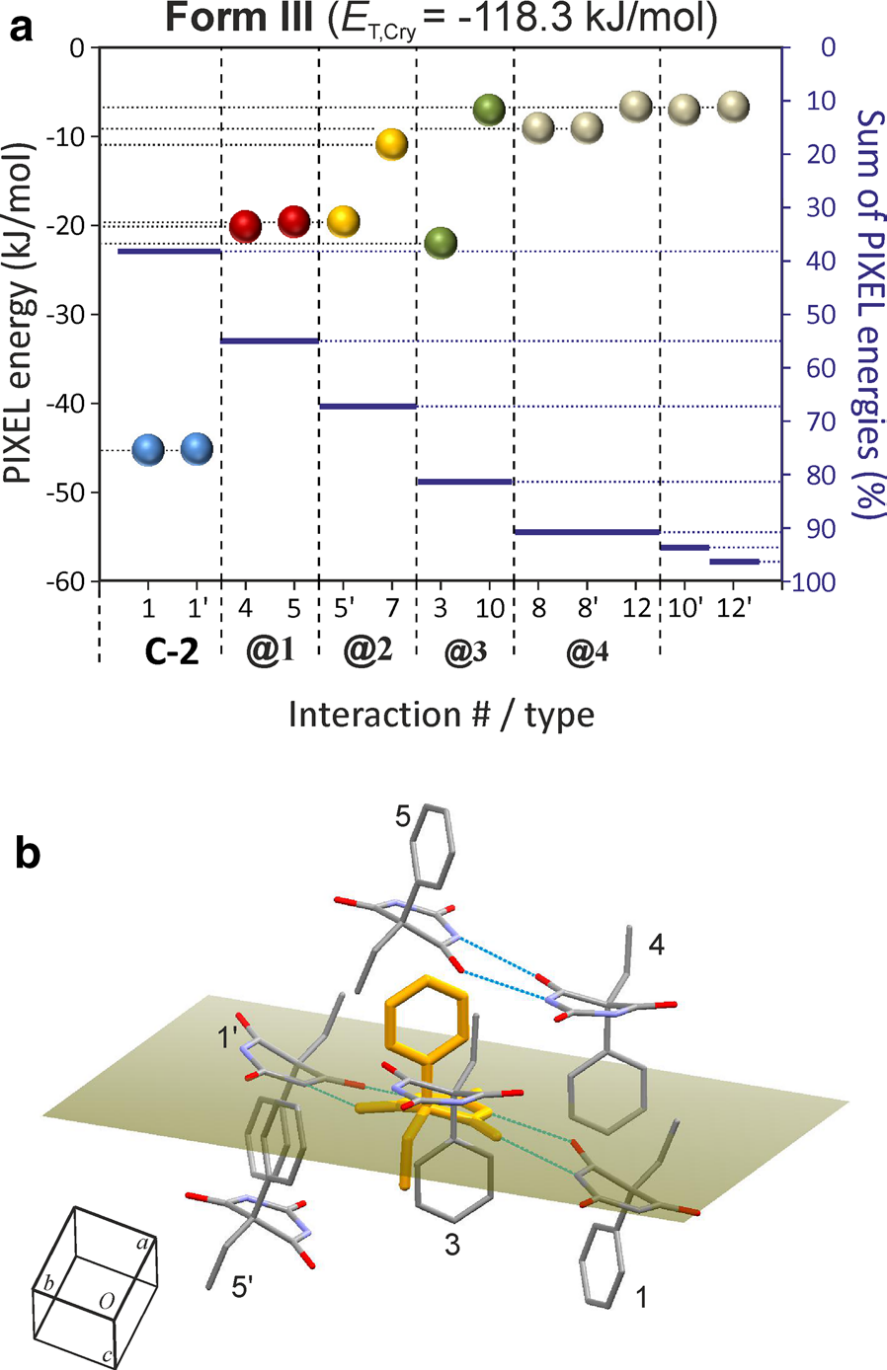
Results of SCDS-PIXEL calculations for polymorph **III**. **a** Interaction energies, represented by balls, are separated into internal **C-2** interactions (*blue*) and chain–chain interactions (*highlighted* **@1**, *red*; **@2**, *orange*; **@3**, *green*). The *horizontal bars* indicate cumulative PIXEL energies (summation from left to right) relative to the *E*_T,Cr*y*_ (scale on the *right-hand side*). **b** The six most important pairwise interactions involving a central molecule (*orange*). The mean plane of the pyrimidine ring of the central molecule is drawn, H atoms are omitted for clarity and H-bonds are indicated by *blue lines*

**Fig. 7:**
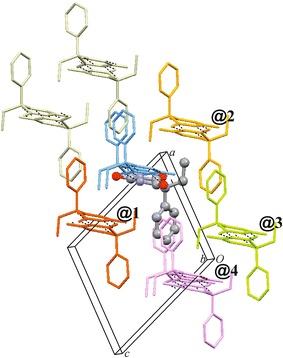
Packing diagram of polymorph **III**, showing interactions of a selected Pbtl molecule (drawn in ball-and-sticks-style) within the same **C-2** chain (*blue*) and with molecules belonging to four neighbouring chains (**@1**–**@4**; see Fig. 6). Together, these interactions account for 91 % of *E*_T,Cry_

The two internal **C-2** interactions account for approximately 38 % of *E*_T,Cry_ of −118.3 kJ mol^−1^, and the interactions with molecules belonging to four neighbouring chains **@1** (2 pairwise interactions), **@2** (2), **@3** (2) and **@4** (3) account for 17, 13, 12 and 11 %, respectively, of *E*_T,Cry_ (Figs. 6, 7). This situation differs somewhat from the packing of **C-1** chains in **X** which is dominated by just two chain–chain interactions (**@1**, **@2**) which contribute 40 % of *E*_T,Cry_.

### HBS types **L-3** + **C-2**: polymorph **I**

The crystal structure of form **I** (space group *P*2_1_/*c*) contains three independent molecules, labelled A–C. A and B molecules are linked into an **L-3** layer (Fig. 2). This layer consists of **C-2** chains, formed exclusively by A molecules, and bridging B molecules. The **L-3** structures lie parallel to (010) and alternate with stacks of **C-2** chains composed of C molecules (Additional file 1: Fig. S4). The two distinct **C-2** chains formed by A and C molecules differ in that the former (as part of a **L-3** layer) possess glide symmetry, whereas the latter contain inversion centres (Additional file 1: Fig. S5).

**Fig. 8:**
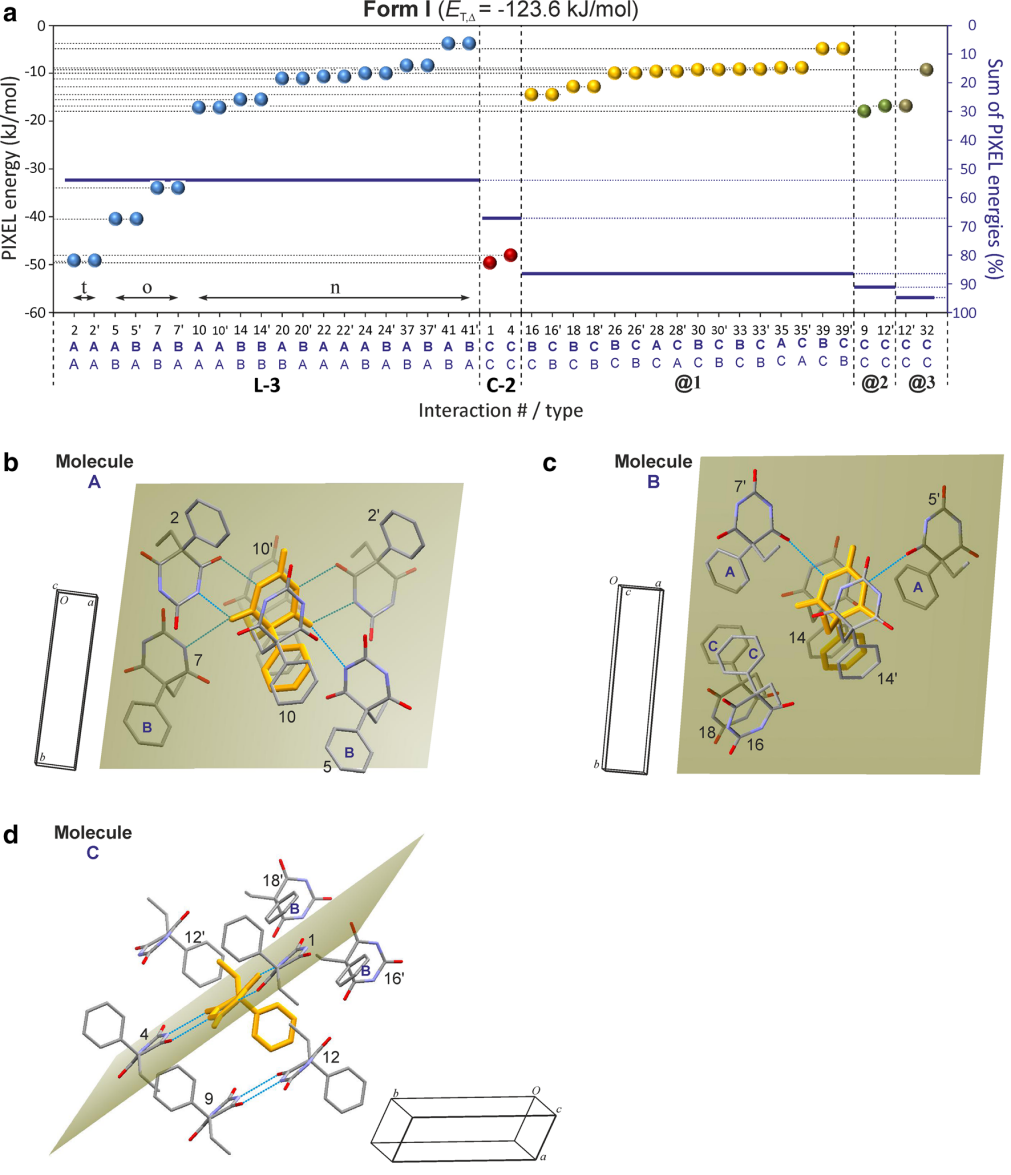
Results of SCDS-PIXEL calculations for polymorph **I**. **a** Interaction energies, represented by balls, are separated into internal **L-3** (*blue*) interactions, internal **C-2** (*red*) interactions, interactions between a **L-3** layer and a stack of **C-2** chains (@1, *orange*) and interactions between neighbouring **C-2** (**@2**, *green*; **@3**, *beige*). The *horizontal bars* indicate cumulative PIXEL energies (summation from left to right) relative to the *E*_T,Cr*y*_ (scale on the *right-hand side*). **b**–**d** A central molecule A, B or C (*coloured orange*) and neighbouring molecules involved in six (**b**, **c**) or seven (**d**) pairwise interactions (see Additional file 1: Tables S1–S3). The mean plane of the pyrimidine ring of the central molecule is drawn, H atoms are omitted for clarity and H-bonds are indicated by *blue lines*

The energy associated with the centrosymmetric t-interaction between A molecules is −49.2 kJ mol^−1^ (#2/2′) and energies of −40.5 and −34.0 kJ mol^−1^ (5/5′ and 7/7′) are calculated for the o-interactions between A and B molecules (Fig. 8). Within an **L-3** layer, the strongest non-H-bonded AA interactions of −17.2 kJ mol^−1^ (#10/10′), between neighbouring **C-2** subunits (related by a [001] translation), and the strongest BB interactions of −15.5 kJ mol^−1^ (#14/14′) each involve relatively large *E*_D_ contributions. There are another eight intra-**L-3** contacts with energies between −11.1 and −8.4 kJ mol^−1^. The energies for the t-connections of the **C-2** chain of molecule C, −49.7 and −48.1 kJ mol^−1^, are very similar to the corresponding values for the **C-2** chains formed by A molecules and in polymorph **III**.

**Fig. 9:**
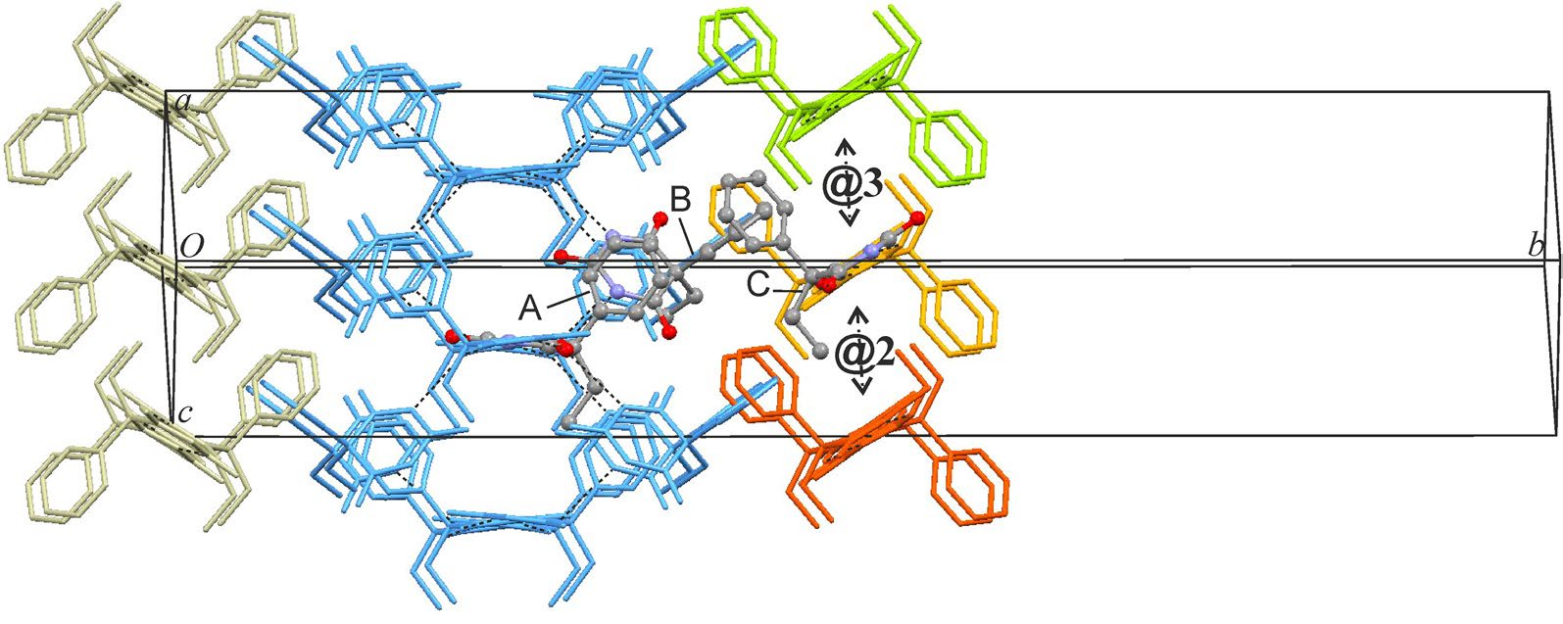
Packing diagram of polymorph **I**. One selected molecule of each type of A, B and C is drawn in ball-and-sticks-style. Together the internal **L-3** (*blue*) and **C-3** (*orange*) interactions account for 67 % of *E*_T,Σ_. Interactions between **L-3** and **C-3** chains (@1) account for 19 % and interactions between neighbouring **C-3** chains (**@2**, **@3**) for 9 % of *E*_T,Σ_

Internal H-bond and non-H-bond interactions of the **L-3** layer account for 54 % and internal **C-2** chain interactions of C molecules account for 13 % of *E*_T,Σ_. Contacts between **L-**3 layers (molecules A + B) and **C-2** stacks (molecule C) contribute 19 % to *E*_T,Σ_ (**@1**), and the contacts **@2** and **@3** between neighbouring **C-2** chains contribute 5 and 4 %, respectively (Figs. 8, 9). Due to their fundamentally different environments and different involvement in N–H···O=C bonds, the three independent molecules also differ substantially in their PIXEL energy sums: 143.1 kJ mol^−1^ (A), −103.8 kJ mol^−1^ (B) and −122.9 kJ mol^−1^ (C).

### HBS types **L-3** + **C-2**: polymorph **II**

Polymorph **II** (space group *P*$\overline{1}$) is a Z′ = 3 structure whose molecules A and B are linked into an **L-3** layer, whilst C-type molecules form a **C-2** chain, and it exhibits a very close 2D packing similarity with polymorph **I** [26]. In fact, the only fundamental difference between these two modifications is the symmetry of the **C-2** chain formed by the respective A-type molecules (**I**: glide symmetry, **II**: inversion; see Additional file 1: Fig. S4).

The comparison of interaction energy diagrams (Additional file 1: Fig. S7; see also Tables S1–S6) shows that this packing similarity results in a striking similarity of corresponding pairwise interaction energies. Therefore, the general assessment of relative energy contributions attributable to **L-3** and **C-2** units and to their packing in polymorph **I** (previous section) is also valid for polymorph **II**.

### HBS type **C-3**: polymorph **V**

**Fig. 10:**
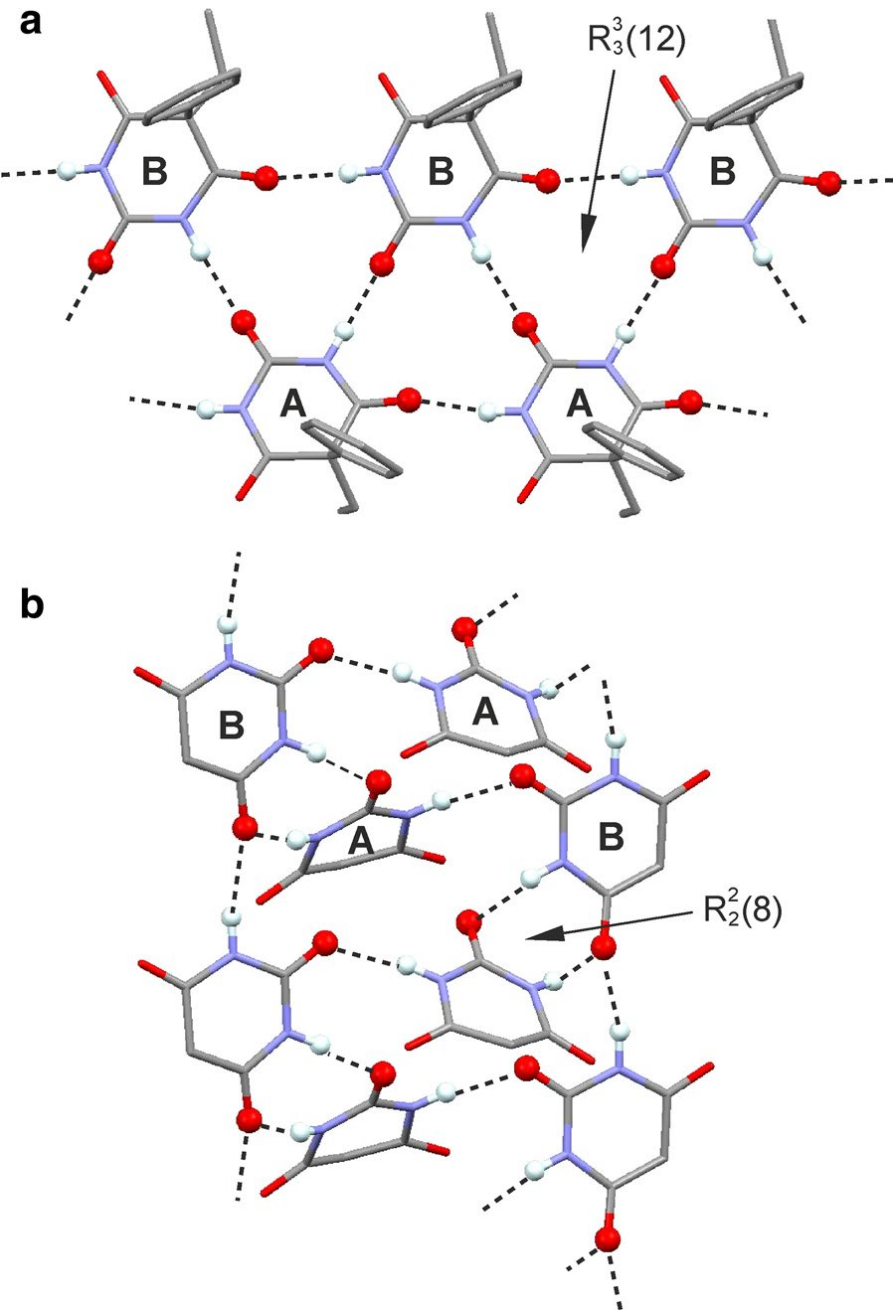
N–H···O=C bonded tapes **C-3** in polymorph **V** (**a**) and **C-5** in polymorph **VI** (**b**). Ethyl and phenyl groups are omitted for clarity. Hydrogen bonds are drawn as *dashed lines*; O and H atoms engaged in H-bond interactions are drawn as balls

Williams [36] reported space group and unit cell data for polymorph **V** which indicated a crystal structure with two independent molecules, and these data are consistent, after unit cell transformation, with those of the full crystal structure analysis carried out by us (see footnote *a* of Table 3). Form **V** has the space group symmetry *P*2_1_/*c* and contains two independent molecules, labelled A and B. It contains N–H···O=C bonded **C-3** tapes (Fig. 10) which are arranged parallel to [010].

Each molecule forms o-connections to four neighbouring molecules. A and B molecules are linked into separate H-bonded strands with translation symmetry, which are offset against one another by one half of a translation period. The linkage between the two parallel strands via N–H···O=C bonds results in fused ${\text{R}}_{3}^{3} \left( {12} \right)$ rings. Although A and B molecules are crystallographically distinct, they are topologically equivalent in the context of the (uninodal) **C-3** structure.

**Fig. 11:**
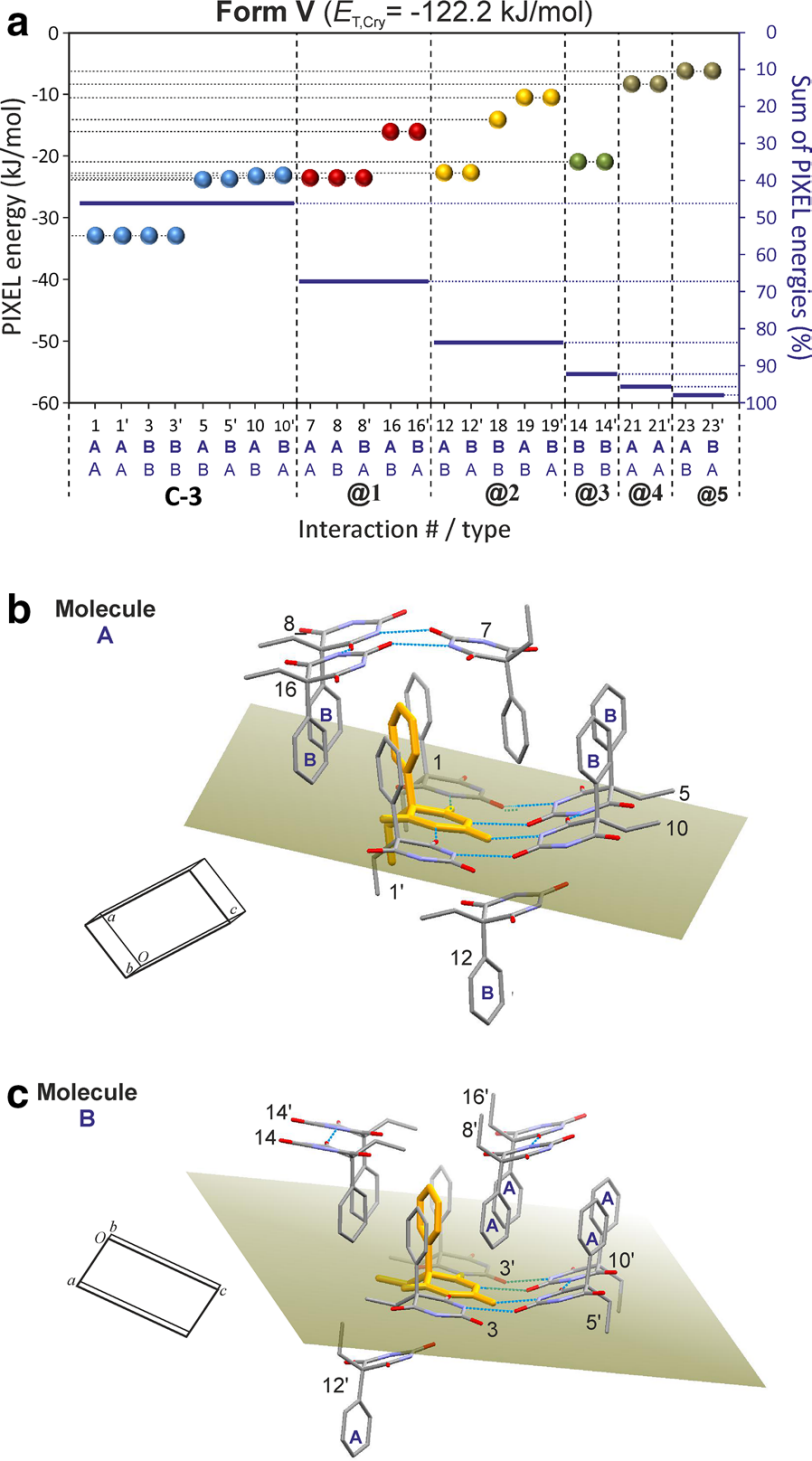
Results of SCDS-PIXEL calculations for polymorph **V**. **a** Interaction energies, represented by balls, are separated into internal **C-3** interactions (*blue*) and interactions between neighbouring **C-3** tapes (*highlighted* **@1**, *red*; **@2**, *orange*; **@3**, *green*). The *horizontal bars* indicate cumulative PIXEL energies (summation from left to right) relative to the *E*_T,Cr*y*_ (scale on the *right-hand side*). A central molecule A (**b**) or B (**c**) (*coloured orange*) and neighbouring molecules involved in eight (**b**) or nine (**c**) pairwise interactions (see Additional file 1: Tables S8 and S9). The mean plane of the pyrimidine ring of the central molecule is drawn, H atoms are omitted for clarity and H-bonds are indicated by *blue lines*

Interaction energies of −32.9 kJ mol^−1^ were obtained both for the o-interactions between A-type molecules (#1/1′) and the analogous interactions between B-molecules (#2/2′). Considerably lower stabilisation effects of −23.8 and −23.2 kJ mol^−1^ result from the o-interactions (#5/5′ and #10/10′) between A and B strands, which is the result of higher (by 9.9–6.4 kJ mol^−1^) dispersion terms. Two H-bonded molecules belonging to different strands have fewer van der Waals interactions with one another than two H-bonded molecules within the same strand (Fig. 11b, c). Moreover, the PIXEL energies of the o-connections #5/5′ and #10/10′ are very similar to those of seven non-H-bond interactions (#7, #8/8′, #12/12′, #14/14′; −23.5 to −20.9 kJ mol^−1^). Each of the latter involves extensive van der Waals contacts (*E*_D_ = −21.9 to −30.7 kJ mol^−1^) which compensate for the lower *E*_C_ contribution in the absence of any N–H···O=C bonding (Additional file 1: Tables S8 and S9). The interactions #12/12′ contain a single contact (mol. B)(CH_2_)C–H···O(mol. A) in which the C2 carbonyl group of molecule A is engaged (H···O 2.58 Å, CHO = 143°), but the associated Coulombic contribution (−11.7 kJ mol^−1^) is less stabilising than *E*_D_ (−28.4 kJ mol^−1^).

**Fig. 12:**
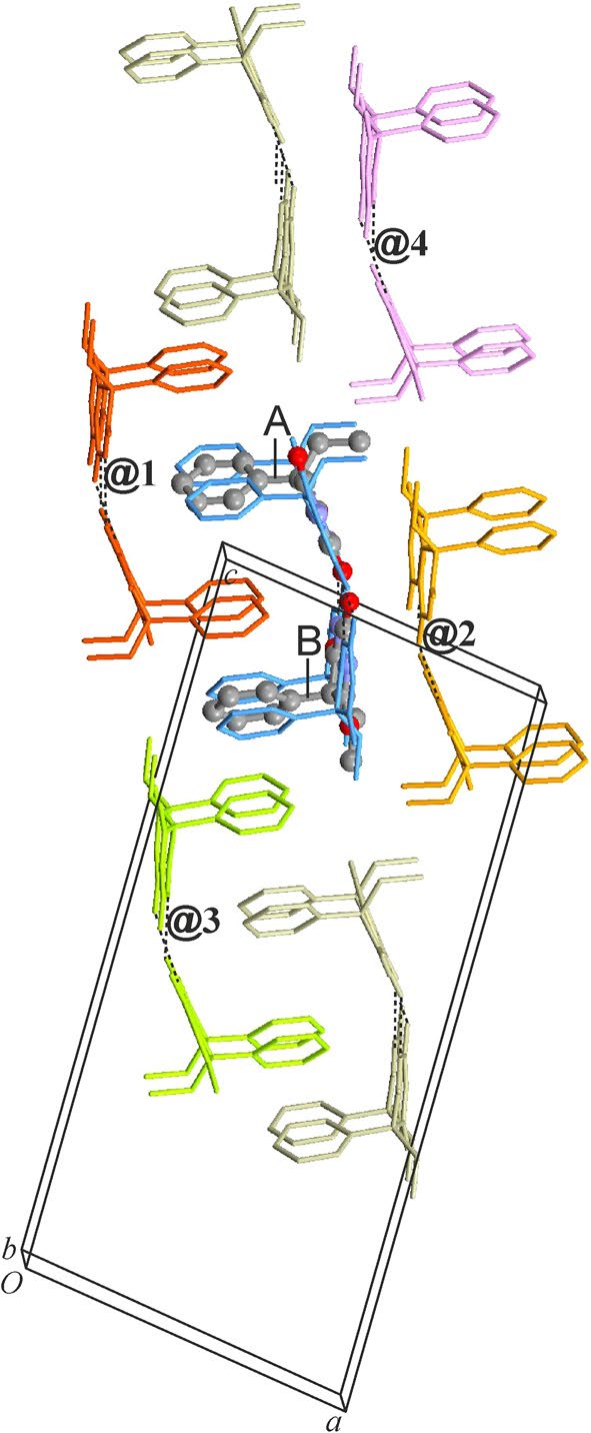
Crystal packing of polymorph **V**. Interactions of selected A and B molecules (drawn in ball-and-sticks-style) within the same **C-3** chain (*blue*) and with molecules belonging to four neighbouring chains (**@1**–**@4**; see Fig. 11). Together, **C-3** hydrogen bonding and the **@1** and **@2** chain stacking interactions account for 84 % of *E*_T,Cry_

The sum of all pairwise interaction energies involving molecule A is 6.5 kJ mol^−1^ higher than the corresponding sum for molecule B. This reflects somewhat different packing environments which are associated with different molecular conformations (see below). Internal **C-3** interactions account for 46 % of *E*_T,Cry_. The **C-3** tapes are arranged in centrosymmetric pairs (**@2**, see Fig. 12) in such a way that the pyrimidine rings of the two tapes are somewhat offset against one another, the ethyl groups are oriented towards the centre of the centrosymmetric unit and the phenyl rings are oriented in the opposite direction. Other centrosymmetric pairs of **C-3** chains result in the mutual antiparallel interdigitation of sets of phenyl groups (**@1**, **@3**). The chain–chain interactions involve either three (**@1**) or two (**@2** and **@3**) of the most stabilising non-H-bond interactions mentioned above (see Fig. 11a). The chain–chain interactions **@1**, **@2** and **@3** account for 21, 16 and 9 %, respectively, of *E*_T,Cry_. This means that 84 % of the stabilisation of the lattice is derived from columnar stacks of **C-3** tapes parallel to [001] which involve the interactions **@1** and **@2** (Fig. 12).

### HBS type **C-5**: polymorph **VI**

Polymorph **VI** has the space group symmetry *P*2_1_/*n* and contains two independent molecules, labelled A and B. It contains the novel N–H···O=C bonded tape structure **C-5** (see Fig. 2) which possesses 2_1_ symmetry. The two molecule types differ in their H-bond connectivity. Each A molecule forms three o-connections (to two A molecules and one B molecule) and one t-connection (to a second B molecule). Each B molecule forms one o- and one t-connection to A-type molecules (Fig. 10b).

The presence of two parallel strands of H-bonded molecules is reminiscent of the **C-3** tape. The **C-5** type displays an unusual asymmetric ${\text{R}}_{2}^{2} \left( 8 \right)$ ring due to N–H···O=C bonds involving the C2 carbonyl function of molecule B and the C4 carbonyl function of molecule A. The energy contribution of −46.5 kJ mol^−1^ associated with this asymmetric t-connection (#1/1′) is very similar to the corresponding values obtained for the symmetric t-connections in forms **I**, **II**, **III** and **X**. The PIXEL energy calculated for the o-connections between A molecules which are related by a translation along [010] (#3/3′; −34.4 kJ mol^−1^) is similar to energies obtained for the analogous interactions in polymorph **V** (#1/1′, #3/3′). The interaction energy for the second set of o-connections (#5/5′) in the **C-5** tape is somewhat higher, −28.4 kJ mol^−1^. In addition to the two o- and four t-connections, the **C-5** tape contains six non-H-bond interactions with PIXEL energies between −13.9 and −8.3 kJ mol^−1^. Altogether, the internal interactions of the **C-5** tape account for 63 % of *E*_T,Cry_.

**Fig. 13:**
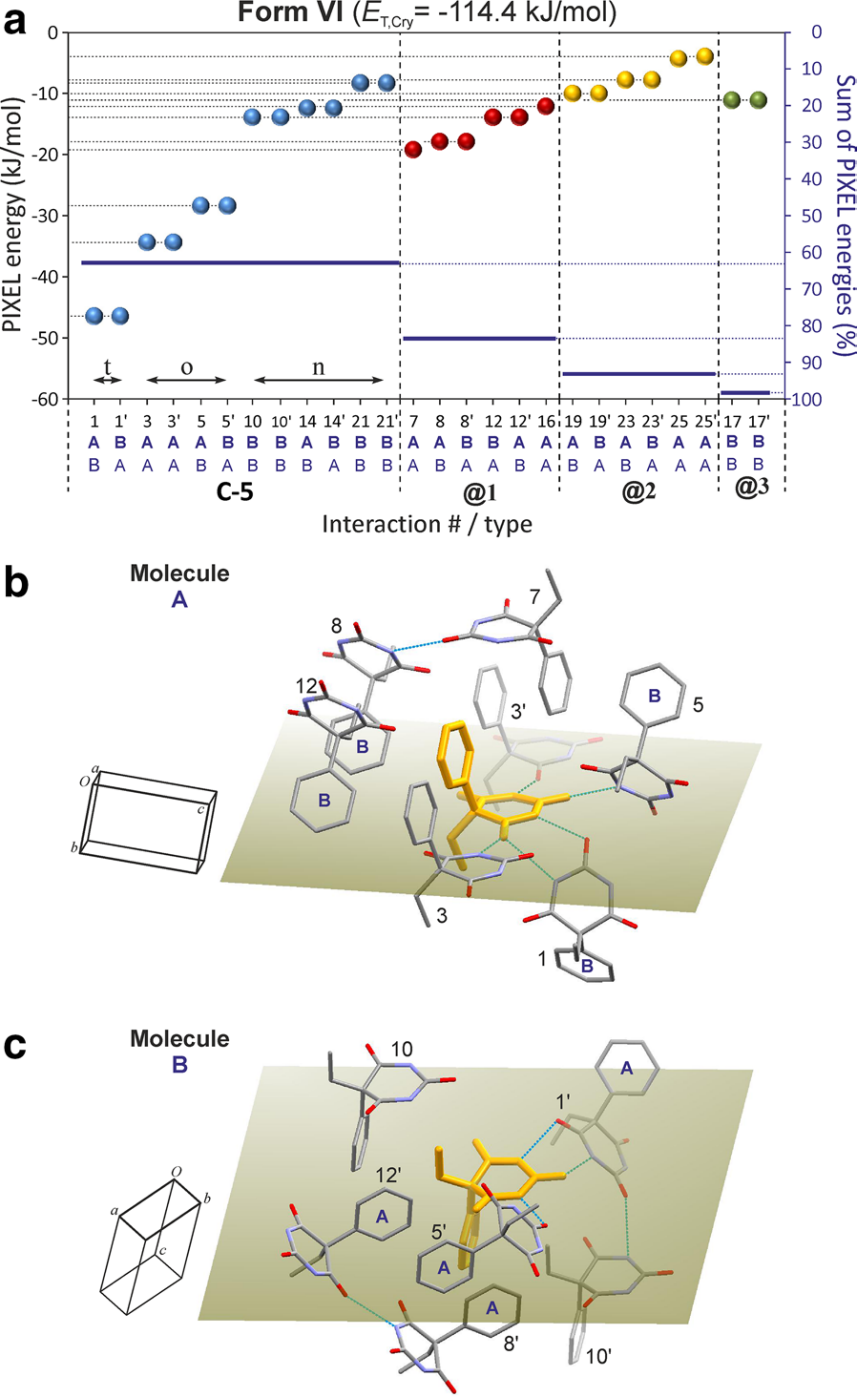
Results of SCDS-PIXEL calculations for polymorph **VI**. **a** Interaction energies, represented by balls, are separated into internal **C-5** interactions (*blue*) and interactions between neighbouring **C-5** tapes (**@1**, *red*; **@2**, *orange*; **@3**, *green*). Internal **C-5** interactions are labelled t (two-point H-bonded), o (one-point H-bonded) and n (non-H-bonded). The *horizontal bars* indicate cumulative PIXEL energies (summation from left to right) relative to the *E*_T,Cr*y*_ (scale on the *right-hand side*). A central molecule A (**b**) or B (**c**) (*coloured orange*) and neighbouring molecules involved in seven (**b**) or six (**c**) pairwise interactions (see Additional file 1: Tables S10 and S11). The mean plane of the pyrimidine ring of the central molecule is drawn, H atoms are omitted for clarity and H-bonds are indicated by *blue lines*

**Fig. 14:**
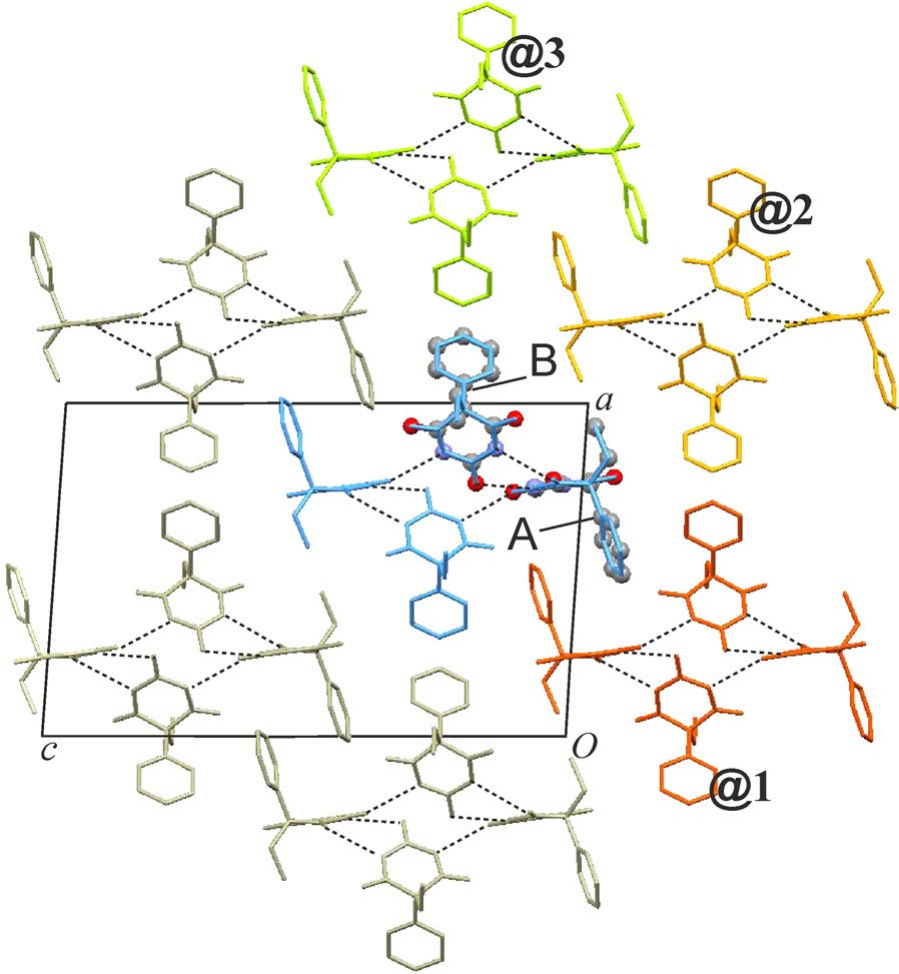
Crystal packing of polymorph **VI**. Interactions of selected A and B molecules (drawn in ball-and-sticks-style) within the same **C-5** chain (*blue*) and with molecules belonging to three neighbouring chains (**@1**–**@3**; see Fig. 13). Together, **C-5** hydrogen bonding and **@1** chain stacking account for 84 % of *E*_T,Cry_

The six strongest external interactions (#7, #8/8′, #12/12′, #18; −19.2 to −12.1 kJ mol^−1^) all involve molecules which belong to a single neighbouring **C-5** tape (**@1**; see Figs. 13a, 14). Each of these molecule–molecule interactions is dominated by the *E*_D_ term as a result of extensive van der Waals contacts, mainly between phenyl groups. In the structure of polymorph **VI**, each instance of **C-5** is surrounded by six other **C-5** tapes (three symmetrical interaction pairs, **@1**, **@2**, **@3**; Fig. 14). The chain–chain interaction **@1** defines, together with the internal **C-5** interactions, the packing within $\left( {10\overline{1} } \right)$ planes which accounts for 85 % of *E*_T,Cry_ and **@1** alone accounts for 21 %. Interactions **@2** (six molecule–molecule contacts) and **@3** (two molecule–molecule contacts) account for approximately 10 and 5 %, respectively, of the stabilisation energy.

### Molecular geometry

The PIXEL energy (*E*_T,Cry_) is an intermolecular energy derived by integration over the isolated molecule charge densities placed in the crystal structure. The electrostatic contribution (*E*_C,Cry_) is rigorously derived by this procedure and various approximations are used to estimate the polarisation (induction; *E*_P,Cry_), dispersion (*E*_D,Cry_) and repulsion (*E*_R,Cry_) contributions to the intermolecular lattice energy. To make the PIXEL crystal energies of different Pbtl polymorphs comparable with one another, we have estimated the intramolecular energy penalties (∆*E*_intra_) of their experimental conformations (Additional file 1: Table S13) with respect to the global conformational energy minimum. The obtained ∆*E*_intra_ values were then added to the PIXEL energy *E*_T,Cry_.

**Fig. 15:**
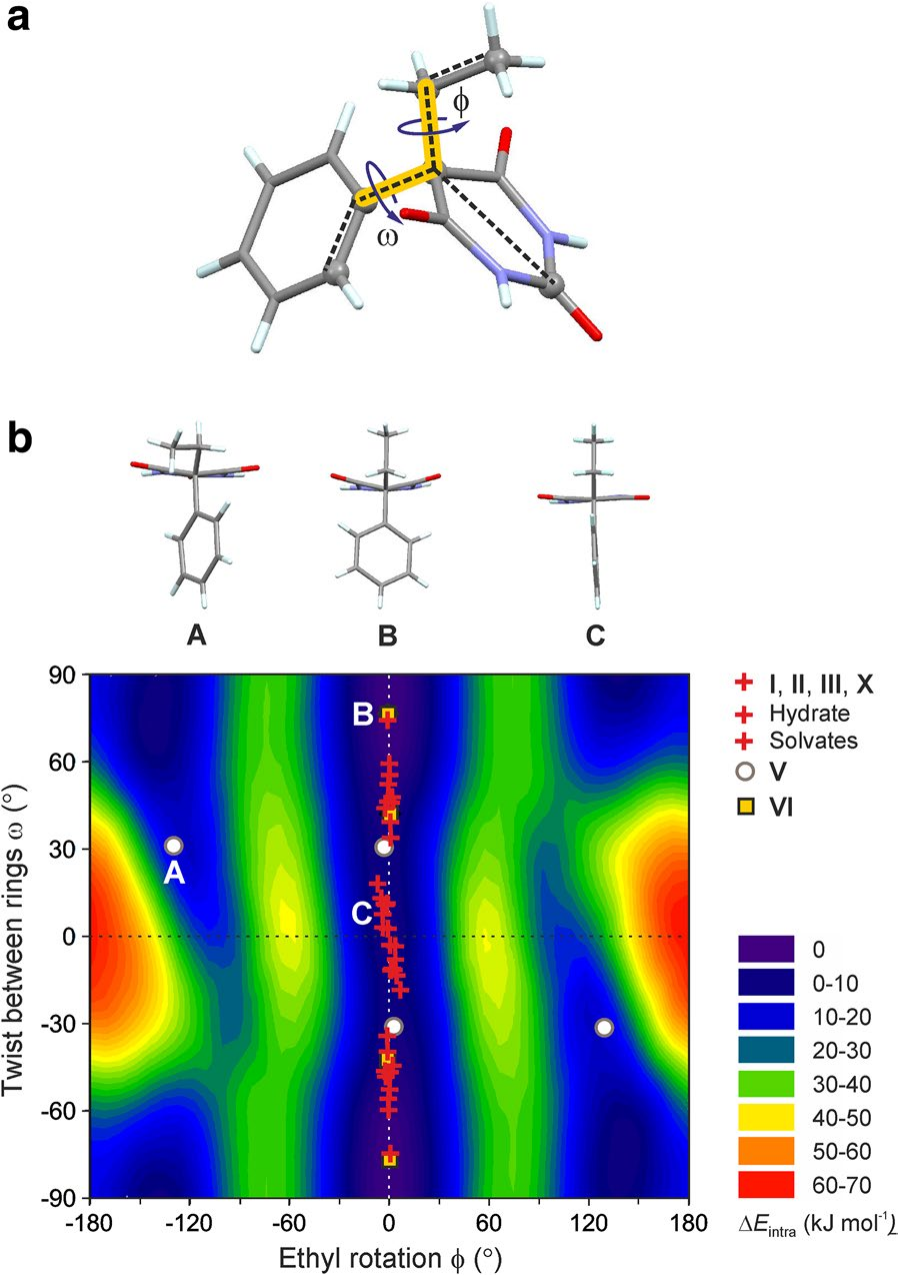
**a** Definition of the torsion angles ϕ and ω used to characterise the molecular geometry of Pbtl. **b** Conformational energy surface of the Pbtl molecule with respect to ϕ and ω, calculated at the MP2 level of theory with the 6-31G(d,p) basis set, with the rest of the molecule optimised in 30° intervals of ϕ and ω. The data points (ϕ, ω)/(−ϕ, −ω) represent the experimental torsion angles in crystal forms of Pbtl, all of which are centrosymmetric. A, B and C are examples of characteristic conformations

The geometry of a Pbtl molecule can be characterised by two parameters, the torsion angle ϕ describing the ethyl rotation and the twist angle ω between the phenyl and pyrimidine rings [42] (Fig. 15a). The ϕ values for all previously reported experimental conformations lie within the narrow range of 0° ± 5°, indicating that the ethyl orientation perpendicular to the pyrimidinetrione ring might be the preferred one in the solid state of Pbtl. At the same time there is a wide variation in the corresponding ω angles from 0° to 75°, which is in agreement with the free rotability of the phenyl group as derived from energy scans for an isolated molecule in the gas phase.

Like all the previously reported Pbtl forms, the conformations of molecule A of polymorph **V**, (ϕ, ω) = (−3°, 31°) and both independent Pbtl molecules of polymorph **VI**, A: (ϕ, ω) = (−1°, 77°) and B: (ϕ, ω) = (1°, 42°) are located in the global energy minimum ‘valley’ (Fig. 15b). The geometry of molecule B of **V**, (ϕ, ω) = (−129°, 31°), is unique in that it can be assigned to the second (local) energy minimum rather than the global energy minimum. A conformational change from the conformer of molecule B to that of molecule A would involve a rotation of the ethyl group (ϕ) by approximately 120° and require approximately 20 kJ mol^−1^. The fact that modification **V** was obtained only from the melt or by sublimation, but never from solution crystallisation experiments, may indicate that a conformation related to the global energy minimum ‘valley’ is preferred in solution.

### Comparison of **IV**, **V** and **X** with previous crystal structure predictions

Pbtl was used by Day et al. [42] as a model flexible molecule in a structure prediction study. 72 structures within 5 kJ mol^−1^ of the global minimum were identified as possible candidates for new polymorphs (in addition to the previously published forms **I**–**III**). Six additional Z′ = 2 candidate structures for polymorph **V** were proposed because they matched the original space group symmetry *P*2_1_/*c* and the reduced cell (*a* = 12.66, *b* = 6.75, *c* = 26.89 Å; β = 99.9°) of Williams’ [36] original cell (*a* = 12.66, *b* = 6.75, *c* = 27.69 Å; β = 106.9°). However, we note that the $\left( {100 0\overline{1} 0 \overline{1} 0\overline{1} } \right)$ transformation involved in this unit cell reduction implies a simultaneous transformation of the space group symmetry from *P*2_1_/*c* to *P*2_1_/*n*. Using the program *XPac* [41, 50], we have compared the new structure models for polymorphs **V**, **VI** and **X** with the 78 theoretical Pbtl structures proposed by Day et al. [42].

**Fig. 16:**
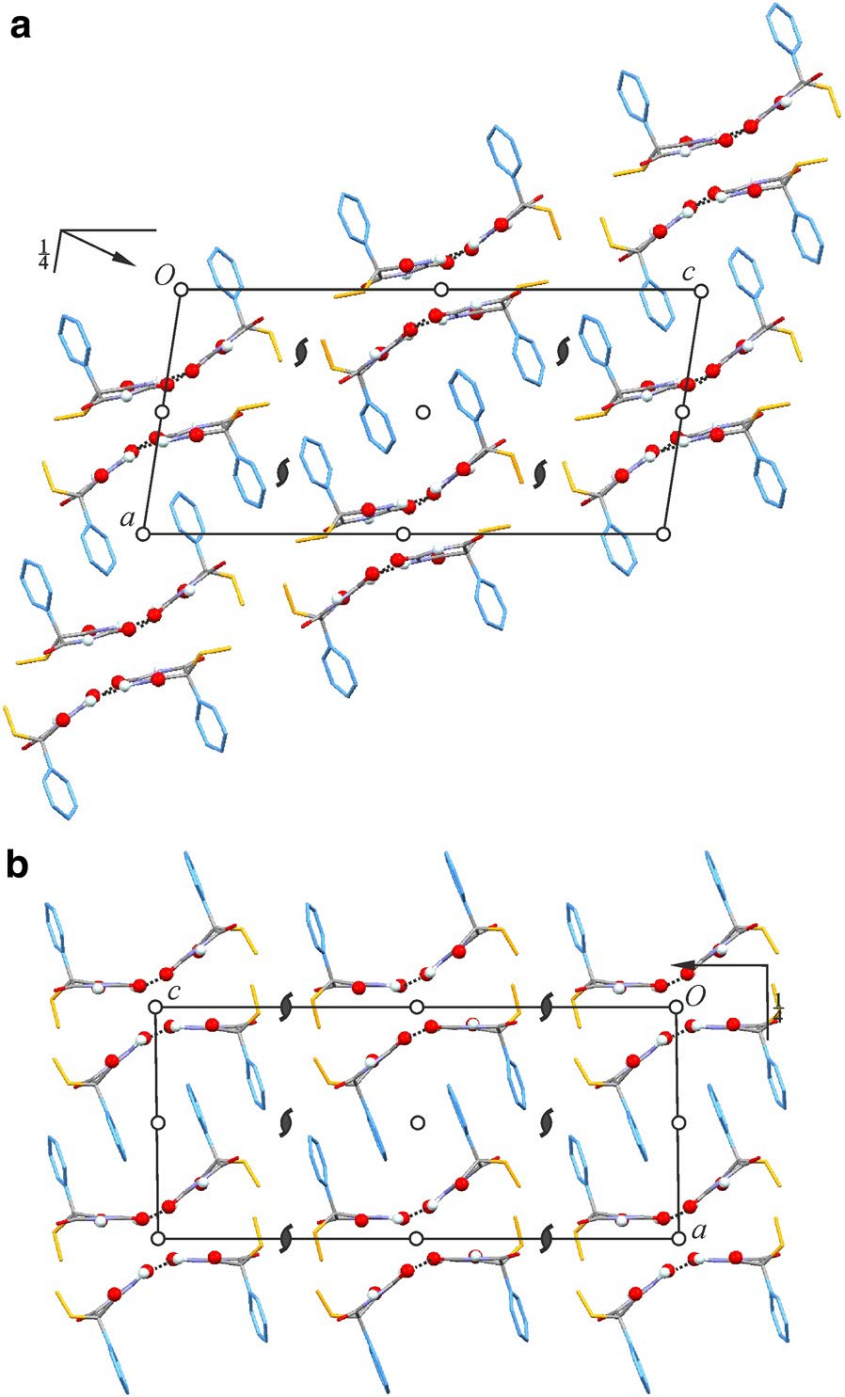
**a** Crystal structure of form **V** of Pbtl (space group *P*2_1_/*n*) and **b** the closest predicted structure for form **V** (space group setting *P*2_1_/*c*) from Ref. [42]. Each structure is viewed along the *b*-axis, the direction of translation of its **C-3** chains. Ethyl and phenyl groups are *coloured orange and blue*, respectively, and O and H engaged in N–H···O interactions are shown as balls; other H atoms are omitted for clarity. Note the fundamental differences in the packing of neighbouring *ab* planes composed of **C-3** chain pairs

There is no complete 3D match for the experimental structure of **V**, but one of the Z′ = 2 candidates for form **V** (#6) with an energy difference from the global minimum of 7.71 kJ mol^−1^ (see Table 2 of Ref. [42]) displays certain features which are reminiscent of the experimental structure of **V** (Additional file 1: Fig. S8). Both structures contain centrosymmetric pairs of **C-3** chains (propagating along [010]) which are arranged into stacks along the *a*-axis in such a way that phenyl groups belonging to neighbouring chain pairs interdigitate (Fig. 16). However, they differ fundamentally in the packing mode between adjacent stacks of H-bonded chains. The molecular conformations (ϕ, ω) = (1°, −21°) and (5°, 23°) for this theoretical structure are both well within the “valley” of low-energy conformations close to ϕ = 0°, whereas in the experimental structure one molecule shows an atypical ethyl rotation with ϕ = −129° (see Fig. 15).

No close match was found for form **VI**, and it seems that its unique **C-5** chain does not occur in any of the theoretical structures. However, there is a very close 3D match between the derived structure model for polymorph **X** (Table 3) and a theoretical structure (#72; reported in *I*2/*a*; transformed *C*2/*c* unit cell: *a* = 12.91 Å, *b* = 20.26 Å, *c* = 10.34 Å; β = 115.3°). An *XPac* comparison based on geometrical parameters derived from complete sets of non-H atoms gives a low dissimilarity index, *x* = 5.2 (see Additional file 1: Fig. S9).

## Discussion

The PIXEL energies for all symmetrical (**C-1**, **C-2**, **L-3**) and asymmetrical (**C-5**) t-connections in Pbtl polymorphs lie between −45.4 and −49.2 kJ mol^−1^ (Table 4). The reason for this relatively narrow range is that the rigid ${\text{R}}_{2}^{2} \left( 8 \right)$ ring geometry permits only small variations in van der Waals interactions and therefore dispersion contributions. The geometry of an o-connection is much less constrained than that of a t-connection, and the corresponding PIXEL energies (−23.1 to −40.5 kJ mol^−1^) can therefore vary by a wide margin. For example, the stabilisation contribution from the strongest o-connection encountered in this study (#5/5′ in the **L-3** layer of **I**) is 5 kJ mol^−1^ lower than that from the weakest t-connection (#1/1′ in the **C-2** chain of **III**), whereas the four weakest o-interactions in the **C-3** chain of **V** (#5/5′, #10/10′) are only just as stabilising as the three strongest non-H-bond interactions in the same crystal structure (#7, #8/8′) (see Fig. 11a). The implied compensation effect arises from a large variation in the dispersion term (e.g. #10/10′: *E*_D_ = −9.5 kJ mol^−1^ vs. #7: *E*_D_ = −30.7 kJ mol^−1^). The observation that enhanced dispersion contributions can fully compensate for the absence of classical H-bonding contradicts the conventional view that H-bonds always dominate the interaction hierarchy but is consistent with recent analyses of chiral carboxylic acids [8] and primary amines [51].

**Table 4:** Sums of internal energies, *E*_HBS,Σ_ (kJ mol^−1^), from N–H···O=C bonded structures in polymorphs of Pbtl and their origin from different types of interaction

HBS	Form	*N*_HBS_ [*N*_o_, *N*_t_, *N*_n_]	*E*_HBS,Σ_	*E*_T_ range (o)	*E*_T_ range (t)	*E*_n,Σ_
**C-1**	**X**	2 [0, 2, 0]	−47.5		−47.2 to −47.7	
**C-2**	**III**	2 [0, 2, 0]	−45.4		−45.4	
**C-2**	**I** (C)	2 [0, 2, 0]	−48.9		−48.1 to −49.7	
**C-2**	**II** (C)	2 [0, 2, 0]	−46.9		−46.8 to −47.0	
**C-3**	**V**	4 [4, 0, 0]	−56.4	−23.1 to −32.9		
**C-5**	**VI**	6 [2, 1, 3]	−72.0	−28.4 to −34.4	−46.5	−17.3
**L-3**	**I** (A + B)	10 [2, 1, 7]	−100.2	−34.0 to −40.5	−49.2	−38.4
**L-3**	**II** (A + B)	10 [2, 1, 7]	−98.6	−35.1 to −38.2	−45.7 to −47.5	−38.7

Contributions arise from *N*_HBS_ pairwise contacts, of which there are *N*_o_ one-point H-bond connections, *N*_T_ two-point connections and *N*_n_ non-H-bond interactions and ranges of interaction energies *E*_T_ (kJ mol^−1^) for the o- and t-connections involved. *E*_n,Σ_ (kJ mol^−1^) is the sum of all significant (internal) non-H-bonded interaction energies within an HBS (**C-5** and **L-3** only)

The (internal) molecule–molecule interactions within an HBS can be classified as being either H-bonded (via an o- or t-connection) or non-H-bonded. The latter type is relevant for the complex **C-5** tape and **L-3** layer structures where it accounts for a PIXEL energy sum of −17 kJ mol^−1^ (**VI**) and approximately −39 kJ mol^−1^ (**I**, **II**), respectively. The first coordination shell of a molecule is of limited size and usually comprises no more than 14 significant interactions with other molecules. Therefore, the total number *N*_HBS_ of internal (H-bond or non-H-bond) of a central molecule is an important characteristic of an HBS.

The average internal energy contribution (*E*_HBS,Σ_) from a **C-1** or **C-2** loop chain (*N*_HBS_ = 2) is −47 kJ mol^−1^. The analogous PIXEL energy sums for the competing **C-3** (*N*_HBS_ = 4), **C-5** (*N*_HBS_ = 6) and **L-3** (*N*_HBS_ = 9) structures are ≈9, ≈25 and ≈52 kJ mol^−1^, respectively, lower than this **C-1**/**C-2** value. Hence, HBSs containing exclusively t-connections result in the lowest and complex tape or layer structures result in the highest internal stabilisation contributions (Table 4). However, its lower *N*_HBS_ number means that the first coordination shell of a t-connected molecule offers more accessible molecule sites for external interactions than that of an o-connected molecule. Specifically, a molecule in a **C-1** or **C-2** chain can engage in two more significant external interactions with molecules belonging to neighbouring chains than a molecule within a **C-3** chain structure. These additional interactions should easily enable a compensation for the internal advantage of **C-3** over **C1/C-2** (≈9 kJ mol^−1^). Therefore, the comparison of *E*_HBS,Σ_ and *N*_HBS_ values suggests that an HBS with t-connections (**C-1**/**C-2**) should be inherently more favourable than any alternative HBS which is based solely on o-connections (**C-3**). In order for the latter to be a viable competitor, it has to enable a set of significantly more favourable external (packing) interactions in comparison to the former.

**Fig. 17:**
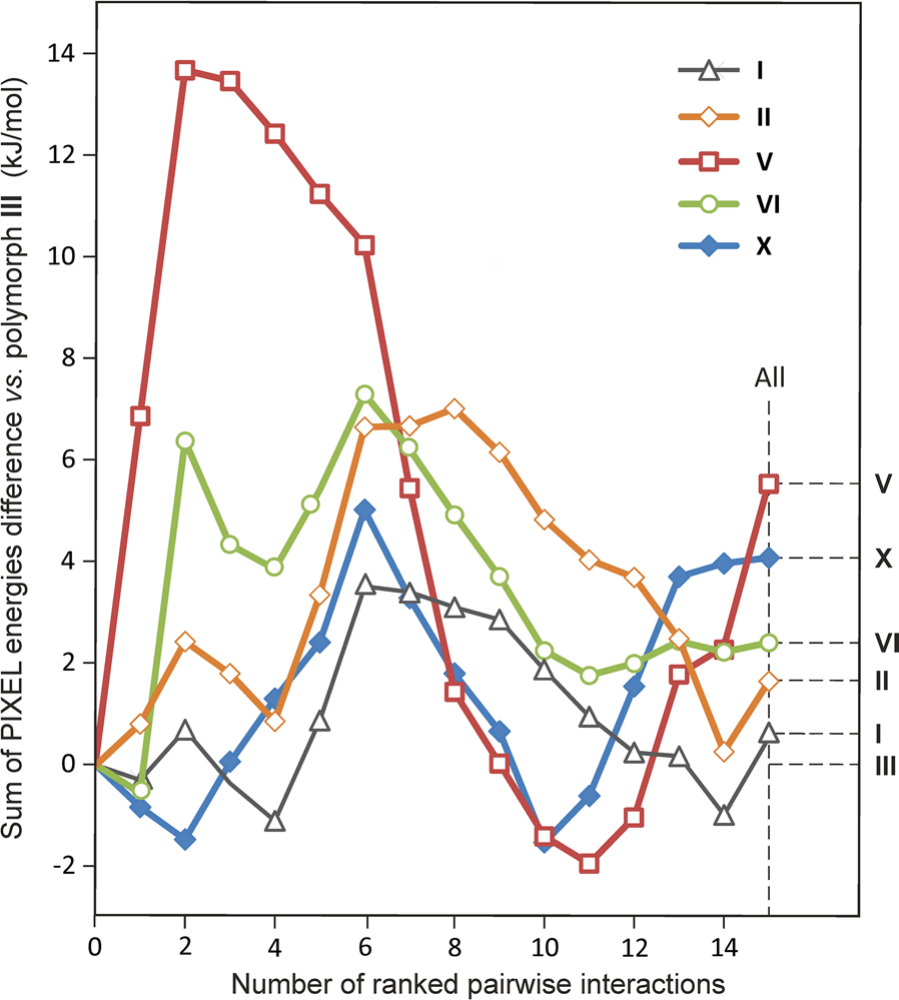
Differences between sums of PIXEL energies, corrected for Δ*E*_intra_, for molecule clusters in polymorphs **I**, **II**, **V**, **VI** and **X** in comparison to the corresponding energy sums calculated for polymorph **III** of Pbtl. For each polymorph, clusters were generated by sequentially adding the 14 most important pairwise energies, ranked in the order of their contribution to the lattice energy from highest to lowest. For each Pbtl polymorph, a *broken horizontal line* indicates the difference to the corrected *E*_T,Σ_ value of polymorph **III**, i.e. (*E*_T,Σ_ + Δ*E*_intra_)^Pbtl^ ^polymorph^ − (*E*_T,Σ_ + Δ*E*_intra_)^**III**^

To analyse the packing effects associated with different HBS types, sums of molecule–molecule interaction energies, corrected for Δ*E*_intra_, have been plotted in a diagram (Fig. 17). For each polymorph, a series of molecular clusters was generated by sequentially adding the 14 most important molecule–molecule interactions (first coordination shell) in descending order of their contributions to the lattice energy. For Z′ > 1 structures (**I**, **V**, **VI**), separate cluster series were generated for independent molecules, whose energy sums were averaged. Each data point in Fig. 17 corresponds to a specific cluster size and represents the difference in energy sums between the indicated polymorph and form **III**. As mentioned above, HBSs dominated by t-connections (**I**–**III**, **X**) are favoured if only the strongest interactions are taken into account.

For all Pbtl polymorphs, the cluster of size 4 contains the complete set of H-bond interactions. Corrected PIXEL energy sums for these clusters in forms **I**, **II** (both *N*_t_ = 4/3) and **III**, **X** (both *N*_t_ = 2) lie within a 2.4 kJ mol^−1^ interval, whereas the corresponding value for polymorph **V** (*N*_t_ = 0) exceeds that of form **III** by more than 12 kJ mol^−1^. The effects of packing multiple **C-5** tapes in form **V** and multiple **C-2** chains in form **III** are such that for each of the next seven highest ranked interactions average PIXEL energies of −17 and −12 kJ mol^−1^, respectively, are obtained. This means that the initial “disadvantage” of **V** has disappeared completely at cluster size 9, and **V** even becomes slightly more favourable than **III** at cluster size 11. If all weak contributions are taken into account, **III** has an overall 5.5 kJ mol^−1^ advantage over **V**. The plot in Fig. 17 illustrates that HBSs based on multiple H-bond connections result in the highest initial stabilisation of small clusters and that HBSs based on o-connections may overcome their inherent “disadvantage” only if they possess superior crystal packing characteristics.

An HBS based on multiple-point connections is more compact and often also of lower dimensionality than an alternative which contains exclusively o-connections (e.g. dimer vs. catemer or **C-1**/**C-2** vs. **C-3**). Therefore, a higher number of theoretical 3D packing options exist for a multiple-point HBS than for a one-point competitor so that it seems likely that more viable crystal packing arrangements would emerge for the former than for the latter. Moreover, compact entities with multiple-point connections may be more likely to exist prior to nucleation and could therefore be kinetically favoured. The domination of the barbiturate set of crystal structures by **C-1** and **C-2** chains (Table 1) could be interpreted in terms of a general preference for HBSs which are based on multiple-point connections.^2^

As discussed above, an interaction between two non-H bonded molecules which involves strong dispersion effects can be as stabilising as an o-interaction with a smaller dispersion contribution (polymorph **V**). The importance of dispersion interactions [51] is not usually recognised in crystal structure discussions, which tend to focus on the interpretation of intermolecular atom–atom distances (with reference to van der Waals radii and standard geometries), for example in terms of conventional or weak hydrogen bonds [52, 53]. The formation of conventional N–H···O=C bonds in barbiturates is largely predictable (but not the exact characteristics of the resulting HBS). By contrast, short intermolecular C–H···O contacts [1], which usually involve a small but significant Coulombic contribution, occur in a rather irregular fashion (see footnotes for Additional file 1: Tables S1–S12). However, in each such case, the crystal contains at least one other molecule–molecule interaction with a lower or only slightly higher PIXEL energy which involves neither an N–H···O=C bond nor a short C–H···O contact. The size of associated *E*_C_ terms (relative to *differences* in *E*_D_ between individual molecule–molecule interactions) as well as the irregularity of their occurrence suggest an opportunistic rather than systematic formation of short C–H···O contacts in Pbtl polymorphs as part of an effort to optimise the stability of the crystal.

The SCDS-PIXEL method allows the comparison of energy sums *E*_T,Σ(A, B,…)_ of interactions originating from the crystallographically distinct molecule types (A, B,…) of a Z′ > 1 structure [54]. In the case of forms **I** and **II**, *E*_T,Σ(A)_ is approximately 20 and 40 kJ mol^−1^ lower than *E*_T,Σ(C)_ and *E*_T,Σ(B)_, respectively (Table 3), which reflects the different involvement of the three independent molecules in o- and t-connections, e.g. [N_o_, N_t_] = [2, 2] (A) or [2, 0] (B) or [0, 2] (C). This means for example that the interactions of molecule B contribute 27.5 % less to the PIXEL energy of the crystal than those of molecule A. A comparison with an overview compiled by Gavezzotti for Z′ = 2 structures (Fig. 7 in Ref. [54]) suggests that the differences in *E*_T,Σ(A, B,…)_ found in Pbtl forms **I** and **II** are unusually large.

In order to demonstrate that the results of the PIXEL calculations presented above are both realistic and consistent, we have attempted to rank the Pbtl polymorphs according to their PIXEL energies and have compared the result with available experimental data. This ranking was based on PIXEL energy sums, *E*_T,Σ_ (Table 3), rather than total PIXEL energies, *E*_T,Cry_, which are not possible to calculate for the Z′ = 3 polymorphs **I** and **II.** Due to the non-additive character of the polarisation contribution, the *E*_T,Σ_ value obtained for each of **III**, **V**, **VI** and **X** is between 1.7 and 3.0 kJ mol^−1^ lower than the corresponding *E*_T,Cry_ value. In order to make the PIXEL crystal energies of all Pbtl forms comparable to one another, experimental molecular conformations (Additional file 1: Table S13) were estimated with respect to the global conformational energy minimum, individual Δ*E*_intra_ values were calculated (Table 3) and added to *E*_T,Cry_. The stability order implied by this procedure is **III** > **I** > **II** > **VI** > **X** > **V**, where the first three forms differ by just 1.7 kJ mol^−1^. This result is in good overall agreement with the findings of a previous experimental study (see Table 3) [26]. Low-temperature (173 K; **II**, **V**, **VI**, **X**) as well as room-temperature (**I**, **III**) structure models were used for our PIXEL calculations. On the basis of a previous report [55] describing two separate PIXEL calculations performed with a room-temperature and a low-temperature structure model of olanzapine, we estimate that the *E*_T,Σ_ values quoted for **I** and **III** in Table 3 should be corrected by approximately −2 % to adjust for different temperatures. Moreover, an optimisation of the model for **X** (derived from the disordered co-crystal structure) would probably have resulted in a slightly lower *E*_T,Σ_.

The Δ*E*_intra_ contributions of the experimental conformations located in the global energy minimum ‘valley’ were estimated to lie within a range of 0.3–8.9 kJ mol^−1^ from the global minimum, with only molecule B of modification **V** adopting a distinct high-energy conformation (17.6 kJ mol^−1^). This higher Δ*E*_intra_ penalty is compensated for by more stable intermolecular interactions.

## Conclusions

There cannot be a straightforward answer to the question whether, for a given group of compounds, an HBS based on multiple-point connections should generally be more favourable than an alternative HBS containing one-point connections (“dimer or catemer?”). Beside geometry restraints and factors such as accessibility and relative strength of H-bond donor and acceptor functions, the competition between alternative HBSs is governed by an interplay between internal energy contributions (from H-bond and non-H-bond molecule–molecule interactions) and stabilisation effects arising from the packing of multiple HBS instances. An HBS based on multiple-point H-bond connections (i.e. a dimer or a **C-1** chain) possesses a more compact architecture than a one-point alternative (i.e. a catemer or a **C-3** tape) and offers a higher number of packing alternatives, which may ultimately result in a higher number of potentially viable low-energy structures. The observation that 60 % of the experimental crystal structures of barbiturates listed in Table 1 contain HBSs which are based exclusively on t-connections may be interpreted in this regard. However, the importance of (external) HBS packing characteristics implies that the competition situation between alternative HBSs can be critically affected by relatively small differences in molecular geometry, for example by the size of the C5 ring substituents in the case of the aforementioned barbiturates.

## Experimental

### Materials

The Pbtl sample used in this study was purchased from Mallinckrodt Chemical Works (U.S.P. XIII Powder, USA) and consisted of a mixture of forms **I** and **II**.

### Preparation of forms **V** and **VI**

Fine needles of **V** were obtained, together with crystals of **II** and **III** from sublimation experiments carried out on a Kofler hot bench, using a setup of two glass slides separated by a 1 cm spacer ring and a sublimation temperature of 135 °C (Additional file 1: Fig. S1). Single crystals of **V**, stored at 5 °C, were stable for at least 2 months, whereas a melt film of form **V** was previously reported to have transformed into either **II** or **III** within hours [26].

Polymorph **VI** was produced, on a hot bench, by the melting and partial dissolution of Pbtl powder immersed in paraffin oil and subsequent crystallisation at 100° C. Prismatic single crystals and spherical polycrystalline aggregates of **VI** were obtained (Additional file 1: Fig. S1).

The identity of the obtained crystals with the Pbtl polymorphs **V** and **VI** was established by comparison of their IR spectra with reference data recorded in a previous study [26] (Additional file 1: Fig. S6).

### Single-crystal X-ray structure analysis^3^

Intensity data were collected, using Cu radiation (**V**) or Mo radiation (**VI**), on an Oxford Diffraction Gemini-R Ultra diffractometer operated by the *CrysAlis* software [56]. The data were corrected for absorption effects by means of comparison of equivalent reflections using the program *SADABS* [57]. The structures were solved using the direct methods procedure in *SHELXS97* and refined by full-matrix least squares on *F*^2^ using *SHELXL97* [58]. Non-hydrogen atoms were refined anisotropically. Hydrogen atoms were located in difference maps and those bonded to carbon atoms were fixed in idealised positions. NH hydrogen atoms were refined with a distance restraint of N–H = 0.88(2) Å. In the case of **V**, the displacement parameters of H atoms were set to 1.2*U*_eq_ (for NH, CH and CH_2_) or 1.5*U*_eq_ (for the CH_3_ group) of the parent N or C atom. In the case of **VI**, these parameters were refined freely. The molecular structures are shown in Additional file 1: Figs. S2 and S3 and the geometric parameters of hydrogen bonds are listed in Table 5. The crystal structure data of polymorphs **V** (CCDC 1035977) and **VI** (CCDC 103598) have been deposited with Cambridge Crystallographic Data Centre.

**Table 5:** Geometric parameters for N–H···O=C bonds

*D*-H···*A*	*d*(*D*-H)/Å	*d*(H···*A*)/Å	*d*(*D*···*A*)/Å	∠(*D*H*A*)/°
Pbtl-**V** (**C-3**)				
(a) N1–H1···O4	0.88(2)	1.92(2)	2.772(5)	164(5)
(b) N3–H3···O2′^ii^	0.878(19)	1.91(2)	2.790(5)	177(5)
(c) N1′–H1′···O4′^iii^	0.87(2)	2.00(2)	2.832(5)	160(4)
(d) N3′–H3′···O2^ii^	0.903(19)	1.99(2)	2.874 (5)	168(5)
Pbtl-**VI** (**C-5**)				
(a) N1–H1···O4^i^	0.887(14)	2.108(15)	2.974(2)	165.2(18)
(b) N3–H3···O2′	0.887(15)	1.966(15)	2.838(2)	167.3(19)
(c) N1′–H1′···O4	0.898(15)	2.052(16)	2.936(2)	168.0(17)
(d) N3′–H3′···O2^v^	0.892(15)	1.969(16)	2.852(2)	170.7(19)

Symmetry transformations: (i) *x*, *y* + 1, *z*; (ii) 1 − *x*, 1 − *y*, 2 − *z*; (iii) *x*, *y* − 1, *z*; (iv) 1 − *x*, 2 − *y*, 2 −*z*; (v) −*x* + 3/2, *y* − ½, −*z* + ½

### Calculation of specific energy contributions

Intermolecular interaction energies were calculated with the semi-classical density sums (SCDS-PIXEL) [37–40] method using the program *OPiX* [59]. Details of these calculations are available in section 5 of Additional file 1. The structure models listed in Table 3 were used, and C–H and N–H distances were re-calculated to standard lengths within *OPiX*. No optimisation of the molecular geometry was performed. An electron density map was calculated on a three-dimensional grid with a step size of 0.08 Å at the MP2/6-31G(d,p) level using Gaussian 09 [60]. A PIXEL condensation factor of 3 was applied, giving superpixels with dimensions 0.24 × 0.24 × 0.24 Å^3^. The calculations yielded interaction energies partitioned into Coulombic, polarisation, dispersion and repulsion terms with an expected accuracy of 1–2 kJ mol^−1^. No more than two independent molecules can be processed in a single *OPiX* procedure. Three separate calculations were therefore carried out for each of the Z′ = 3 forms **I** and **II** in order to obtain a full set of pairwise interaction energies.

### Potential-energy surface scan

The deformation energy for the Pbtl molecule was computed on a 13 × 13 grid, equivalent to a 30° grid spacing for each dihedral angle in the range from 0° to 360° for ϕ and ω, using Gaussian 09 [60]. At each grid point the deformation energy was calculated with the flexible torsions fixed and the rest of the molecule (i.e. all other torsions, angles and bond lengths) optimised at the MP2/6-31G(d,p) level of theory. Additionally, the conformational energy penalties (Δ*E*_intra_) with respect to the global conformational energy minimum were calculated, keeping the experimental ϕ and ω torsions fixed, and the rest of the molecule was minimised using the same method as applied for the grid calculations.

### Analysis and comparison of crystal structure data

The topologies of HBSs (Table 2) were determined and classified with the programs *ADS* and *IsoTest* of the *TOPOS* package [61] in the manner described by Baburin and Blatov [62].

Geometrical comparisons between crystal structures were carried with the program *XPac* [41, 50]. The underlying calculations were based on intermolecular geometrical parameters obtained from all 11 non-H atomic positions of the Pbtl molecule (for details, see Additional file 1: Section 7). In order to minimise effects arising from different molecular conformations, a second set of calculations was performed which was based only on the 1,3,5-pyrimidinetrione unit and the C atoms bonded to ring atom C5.

## Additional file

10.1186/s13065-016-0152-5 Details of the preparation of forms **V** and **VI**, thermal ellipsoid plots for **V** and **VI**, structural information about forms **I**–**III**, IR spectra of **V** and **VI**, details of PIXEL calculations and *XPac* comparisons, derived structure model for **X**.

## Abbreviations

Pbtl: phenobarbital
**I**, **II**, **III**…**XI**: polymorphic forms of phenobarbital
HBS: hydrogen-bonded structure
**C-*****n***, **L-*****n***, **F-*****n***, (*n* = 1, 2, 3…): types of H-bonded structures of barbiturates (Ref. [23])
o-connection: connection of two molecules by a single H-bond interaction
t-connection: connection of two molecules by two H-bond interactions
*N*_o_: number of o-connections per molecule
*N*_t_: number of t-connections per molecule
*E*_C_: PIXEL Coulombic energy term (pairwise interaction)
*E*_P_: PIXEL polarisation energy term (pairwise interaction)
*E*_D_: PIXEL dispersion energy term (pairwise interaction)
*E*_R_: PIXEL repulsion energy term (pairwise interaction)
*E*_T_: total PIXEL interaction energy (pairwise interaction)
*E*_T,Cry_: total PIXEL energy of the crystal
*E*_T,Σ_: sum of all *E*_T_ energies of the crystal
*E*_T,Σ(A or B or C)_: sum of all *E*_T_ energies in a Z′ > 1 structure involving a specific independent molecule (A or B or C)
Δ*E*_intra_: intramolecular energy penalty
*E*_HBS,Σ_: sum of all internal molecule–molecule energy contributions of an HBS
#1, #2, #3…: labels for pairwise interactions between molecules
**@1**, **@2**, **@3**…: labels for pairwise interactions between different H-bonded entities

## Footnotes

1 The carbonyl group at C2 will be referred to as “C2 carbonyl group” and any one of the two topologically equivalent carbonyl groups at C4 or C6 will be referred to as “C4/C6 carbonyl group”.

2 The fact that only 12 of the theoretical low energy structures reported by Day et al. [35] contain **C-1** or **C-2** chains may be due to modelling errors. We note also that 15 of the 72 predicted Pbtl structures contain one NH group which is not engaged in an intermolecular N−H∙∙∙O interaction, a characteristic not encountered in any relevant experimental crystal structures of Pbtl analogues (Table 1).

3 Crystal data for form **V**: C_12_H_12_N_2_O_3_, *M* = 232.24, monoclinic, *a* = 12.7606(12) Å, *b* = 6.7624(5) Å, *c* = 26.847(3) Å, *β* = 98.829(9)°, *V* = 2289.2(4) Å^3^, *T* = 173(2) K, space group *P*2_1_*/n*, *Z* = 8, 7565 reflections measured, 3822 independent reflections (*R*_*int*_ = 0.1439); 3.3° ≤ θ ≤ 65.0° (λ = 1.5418 Å). The final *R*_*1*_ value was 0.0718 (*I* > 2*σ*(*I*)). The final *wR*(*F*^2^) values were 0.1310 (*I* > 2*σ*(*I*)) and 0.1775 (all data). The max. and min. residual densities were 0.23 and −0.22 e Å^−3^, respectively. Crystal data for form **VI**: C_12_H_12_N_2_O_3_, *M* = 232.24, monoclinic, *a* = 14.6701(11) Å, *b* = 6.9000(5) Å, *c* = 23.0308(19) Å, *β* = 94.072(7)°, *V* = 2325.4(3) Å^3^, *T* = 173(2) K, space group *P*2_1_*/n*, *Z* = 8, 9105 reflections measured, 4104 independent reflections (*R*_*int*_ = 0.0499); 3.1° ≤ θ ≤ 25.1° (λ = 0.71073 Å). The final *R*_*1*_ value was 0.0458 (*I* > 2*σ* (*I*)) and the final *wR*(*F*^2^) values were 0.0763 (*I* > 2*σ*(*I*)) and 0.0866 (all data). The max. and min. residual densities were 0.20 and −0.21 e Å^−3^, respectively.

## References

[1] Desiraju GR, Steiner T (1999) Conventional and non-conventional bonds. In: The weak hydrogen bond in structural chemistry and biology. IUCr monographs on crystallography, vol 9. Oxford University Press, Oxford

[2] Beyer T, Price SL (2000). Dimer or catemer? Low-energy crystal packings for small carboxylic acids. J Phys Chem B.

[3] Sanphui P, Bolla G, Das U, Mukherjee AK, Nangia A (2013). Acemetacin polymorphs: a rare case of carboxylic acid catemer and dimer synthons. CrystEngComm.

[4] Gavezzotti A, Filippini G (1995). Polymorphic forms of organic crystals at room conditions: thermodynamic and structural implications. J Am Chem Soc.

[5] Neumann MA, Perrin M-A (2009). Can crystal structure prediction guide experimentalists to a new polymorph of paracetamol?. CrystEngComm.

[6] Chan HCS, Kendrick J, Leusen FJJ (2011). Molecule VI, a benchmark crystal-structure-prediction sulfonimide: are its polymorphs predictable?. Angew Chem Int Ed.

[7] Nyman J, Day GM (2015). Static and lattice vibrational energy differences between polymorphs. CrystEngComm.

[8] Gavezzotti A, Lo Presti L (2015). Theoretical study of chiral carboxylic acids. Structural and energetic aspects of crystalline and liquid states. Cryst Growth Des.

[9] Hisamatsu S, Masu H, Azumaya I, Takahashi M, Kishikawa K, Kohmoto S (2011). U-Shaped aromatic ureadicarboxylic acids as versatile building blocks: construction of ladder and zigzag networks and channels. Cryst Growth Des.

[10] Barnett SA, Hulme AT, Issa N, Lewis TC, Price LS, Tocher DA, Price SL (2008). The observed and energetically feasible crystal structures of 5-substituted uracils. New J Chem.

[11] Florence AJ, Bedford CT, Fabbiani FPA, Shankland K, Gelbrich T, Hursthouse MB, Shankland N, Johnston A, Fernandes P (2008). Two-dimensional similarity between forms I and II of cytenamide, a carbamazepine analogue. CrystEngComm.

[12] Florence AJ, Shankland K, Gelbrich T, Hursthouse MB, Shankland N, Johnston A, Fernandes P, Leech CK (2008). A catemer-to-dimer structural transformation in cyheptamide. CrystEngComm.

[13] Arlin J-B, Price LS, Price SL, Florence AJ (2011). A strategy for producing predicted polymorphs: catemeric carbamazepine form V. Chem Commun.

[14] Arlin J-B, Johnston A, Miller GJ, Kennedy AR, Price SL, Florence AJ (2010). A predicted dimer-based polymorph of 10,11-dihydrocarbamazepine (form IV). CrystEngComm.

[15] Braun DE, McMahon JA, Koztecki LH, Price SL, Reutzel-Edens SM (2014). Contrasting polymorphism of related small molecule drugs correlated and guided by the computed crystal energy landscape. Cryst Growth Des.

[16] Braun DE, Gelbrich T, Kahlenberg V, Tessadri R, Wieser J, Griesser UJ (2009). Conformational polymorphism in aripiprazole: preparation, stability and structure of five modifications. J Pharm Sci.

[17] Nanubolu JB, Sridhar B, Babu VSP, Jagadeesh B, Ravikumar K (2012). Sixth polymorph of aripiprazole: an antipsychotic drug. CrystEngComm.

[18] Delaney SP, Pan D, Yin SX, Smith TM, Korter TM (2013). Evaluating the roles of conformational strain and cohesive binding in crystalline polymorphs of aripiprazole. Cryst Growth Des.

[19] Hursthouse MB, Hughes DS, Gelbrich T, Threlfall TL (2015). Describing hydrogen-bonded structures; topology graphs, nodal symbols and connectivity tables, exemplified by five polymorphs of each of sulfathiazole and sulfapyridine. Chem Cent J.

[20] Gelbrich T, Hursthouse MB (2007). Threlfall TL (2007) Structural systematics of 4,4′-disubstituted benzenesulfonamidobenzenes. 1. Overview and dimer-based isostructures. Acta Crystallogr, Sect B: Struct Sci.

[21] Sanphui P, Rajput L (2014). Tuning solubility and stability of hydrochlorothiazide co-crystals. Acta Crystallogr Sect B: Struct Sci.

[22] Roux MV, Temprado M, Notario R, Foces-Foces C, Emel’yanenko VN, Verevkin SP (2008). Structure-energy relationship in barbituric acid: a calorimetric, computational, and crystallographic study. J Phys Chem A.

[23] Gelbrich T, Rossi D, Häfele CA, Griesser UJ (2011). Barbiturates with hydrogen-bonded layer and framework structures. CrystEngComm.

[24] Gelbrich T, Griesser UJ (2015). Crystal structure of the α-racemate of methohexital. Acta Crystallogr Sect E: Struct Rep Online.

[25] Zencirci N, Gelbrich T, Kahlenberg V, Griesser UJ (2009). Crystallization of metastable polymorphs of phenobarbital by isomorphic seeding. Cryst Growth Des.

[26] Zencirci N, Gelbrich T, Apperley DC, Harris RK, Kahlenberg V, Griesser UJ (2010). Structural features, phase relationships and transformation behavior of the polymorphs I–VI of phenobarbital. Cryst Growth Des.

[27] Abraham A, Apperley DC, Gelbrich T, Harris RK, Griesser UJ (2011). NMR crystallography: three polymorphs of phenobarbital. Can J Chem.

[28] Brandstätter-Kuhnert M, Aepkers M (1962). Molecular compounds, crystalline solid solutions, and new cases of polymorphism in barbiturates. II. Microchim Acta.

[29] Brandstätter-Kuhnert M, Aepkers M (1963). Molecular compounds, crystalline solid solutions, and new cases of polymorphism in barbiturates. III. Microchim Acta.

[30] Brandstätter-Kuhnert M, Aepkers M (1961). Polymorphism of barbiturates by microscopical thermal analysis of two-component systems. Mikroskopie.

[31] Kuhnert-Brandstätter M, Vlachopoulos A (1967). Molecular compounds, crystalline solid solutions, and new polymorphism of barbiturates. IV. Mikrochim Acta.

[32] Zencirci N, Griesser UJ, Gelbrich T, Apperley DC, Harris RK (2014). Crystal polymorphs of barbital: news about a classic polymorphic system. Mol Pharm.

[33] Williams P (1974). Polymorphism of phenobarbitone. II. The crystal structure of modification III. Acta Crystallogr, Sect B: Struct Sci.

[34] Platteau C, Lefebvre J, Hemon S, Baehtz C, Danede F, Prevost D (2005). Structure determination of forms I and II of phenobarbital from X-ray powder diffraction. Acta Crystallogr Sect B: Struct Sci.

[35] Zencirci N, Griesser UJ, Gelbrich T, Kahlenberg V, Jetti RKR, Apperley DC, Harris RK (2014). New solvates of an old drug compound (phenobarbital): structure and stability. J Phys Chem B.

[36] Williams PP (1973). Polymorphism of phenobarbitone: the crystal structure of 5-ethyl-5-phenylbarbituric acid monohydrate. Acta Crystallogr Sect B: Struct Sci.

[37] Dunitz JD, Gavezzotti A (2005). Molecular recognition in organic crystals: directed intermolecular bonds or nonlocalized bonding?. Angew Chem Int Ed.

[38] Gavezzotti A (2007). Molecular aggregation: Structure analysis and molecular simulation of crystals and liquids.

[39] Gavezzotti A (2005). Calculation of lattice energies of organic crystals: the PIXEL integration method in comparison with more traditional methods. Z Kristallogr.

[40] Gavezzotti A (2005). Quantitative ranking of crystal packing modes by systematic calculations on potential energies and vibrational amplitudes of molecular dimers. J Chem Theory Comput.

[41] Gelbrich T, Hursthouse MB (2005). A versatile procedure for the identification, description and quantification of structural similarity in molecular crystals. CrystEngComm.

[42] Day GM, Motherwell WDS, Jones W (2007). A strategy for predicting the crystal structures of flexible molecules: the polymorphism of phenobarbital. Phys Chem Chem Phys.

[43] Groom CR, Allen FH (2014). The Cambridge Structural Database in retrospect and prospect. Angew Chem Int Ed.

[44] Etter MC, MacDonald JC, Bernstein J (1990). Graph-set analysis of hydrogen-bond patterns in organic crystals. Acta Crystallogr Sect B: Struct Sci.

[45] Bernstein J, Davis RE, Shimoni L, Chang N-L (1995). Patterns in hydrogen bonding: functionality and graph set analysis in crystals. Angew Chem Int Ed.

[46] Lewis TC, Tocher DA, Price SL (2004). An experimental and theoretical search for polymorphs of barbituric acid: the challenges of even limited conformational flexibility. Cryst Growth Des.

[47] DesMarteau DD, Pennington WT, Resnati G (1994). Fluorinated barbituric acid derivatives. Acta Crystallogr Sect C: Cryst Struct Commun.

[48] Rossi D, Gelbrich T, Kahlenberg V, Griesser UJ (2012). Supramolecular constructs and thermodynamic stability of four polymorphs and a co-crystal of pentobarbital (nembutal). CrystEngComm.

[49] Gelbrich T, Meischberger I, Griesser UJ (2015). Two polymorphs of 5-cyclohexyl-5-ethylbarbituric acid and their packing relationships with other barbiturates. Acta Crystallogr Sect C: Cryst Struct Commun.

[50] Gelbrich T, Threlfall TL, Hursthouse MB (2012). *XPac* dissimilarity parameters as quantitative descriptors of isostructurality: the case of fourteen 4,5′-substituted benzenesulfonamido-2-pyridines obtained by substituent interchange involving Cf_3_/I/Br/Cl/F/Me/H. CrystEngComm.

[51] Maloney AGP, Wood PA, Parsons S (2014). Competition between hydrogen bonding and dispersion interactions in the crystal structures of the primary amines. CrystEngComm.

[52] Dunitz J (2015). Intermolecular atom–atom bonds in crystals?. IUCrJ.

[53] Thakur TS, Dubey R, Desiraju GR (2015). Intermolecular atom–atom bonds in crystals: a chemical perspective. IUCrJ.

[54] Gavezzotti A (2008). Structure and energy in organic crystals with two molecules in the asymmetric unit: causality or chance?. CrystEngComm.

[55] Bhardwaj RM, Price LS, Price SL, Reutzel-Edens SM, Miller GJ, Oswald IDH, Johnston BF, Florence AJ (2013). Exploring the experimental and computed crystal energy landscape of olanzapine. Cryst Growth Des.

[56] CrysAlis CCD, CrysAlis RED (2003). Oxford Diffraction Ltd.

[57] Sheldrick GM (2007). SADABS. Version 2007/7.

[58] Sheldrick GM (2008). A short history of SHELX. Acta Crystallogr, Sect A: Found Crystallogr.

[59] Gavezzotti A (2007). OPiX: A computer program package for the calculation of intermolecular interactions and crystal energies.

[60] Frisch MJ, Trucks GW, Schlegel HB, Scuseria GE, Robb MA, Cheeseman JR, Scalmani G, Barone V, Mennucci B, Petersson GA (2009). Gaussian 09.

[61] Blatov VA (2006). Multipurpose crystallochemical analysis with the program package topos. IUCr Compcomm Newsl.

[62] Baburin IA, Blatov VA (2007). Three-dimensional hydrogen-bonded frameworks in organic crystals: a topological study. Acta Crystallogr Sect B: Struct Sci.

[63] Roux MV, Notario R, Foces-Foces C, Temprado M, Ros F, Emel’yanenko VN, Verevkin SP (2010). Experimental and computational thermochemical study and solid-phase structure of 5,5-dimethylbarbituric acid. J Phys Chem A.

[64] Bideau JP (1971). Crystal structure of 5-ethyl-5-butylbarbituric acid. C R Acad Sci, Ser C: Sci Chim.

[65] Nichol GS, Clegg W (2005). 5-Butyl-5-ethylbarbituric acid: a phase transition at low temperature. Acta Crystallogr Sect C: Cryst Struct Commun.

[66] Nichol GS, Clegg W (2007). A second *C*2/*c* polymorph of butobarbitone. Acta Crystallogr Sect E: Struct Rep Online.

[67] Bideau JP, Marsau P (1974). 5-Ethyl-5-*n*-pentyl barbituric acid. Cryst Struct Commun.

[68] Craven BM, Vizzini EA (1969). The crystal structures of two polymorphs of 5-ethyl-5-isoamylbarbituric acid (amobarbital). Acta Crystallogr Sect B: Struct Sci.

[69] Jones GP, Andrews PR (1981). Conformations of barbiturates related to pentobarbitone. I. Crystal structure of trans-5-ethyl-5-but-1′-enyl barbituric acid. J Chem Cryst.

[70] Jones GP, Andrews PR (1981). Conformations of barbiturates related to pentobarbitone. III. Crystal structure of 5-ethyl-5-(3′-methylbut-2′-enyl) barbituric acid. J Chem Cryst.

[71] Andrews PR, Jones GP (1981). Conformations of barbiturates related to pentobarbitone. II. Crystal structure of trans-5-ethyl-5-(1′,3′-dimethylbut-1′-enyl) barbituric acid. J Chem Cryst.

[72] Jones GP, Horn E (1986). Conformations of barbiturates related to pentobarbitone. IV. Crystal structure of 5-ethyl,5-(1′,3′-dimethylbut-2′-enyl) barbituric acid. J Chem Cryst.

[73] Smit PH, Kanters JA (1974). The crystal and molecular structure of 5-ethyl-5-(l,3-dimethylbutyl)barbituric acid (α-methylamobarbital). Acta Crystallogr Sect B: Struct Sci.

[74] Escobar C (1975). Molecular and crystal structure of 5,5-diallylbarbituric acid. Acta Crystallogr Sect B: Struct Sci.

[75] Rae AD (1975). Polymorph of 5-allyl-5-isopropylbarbituric acid (aprobarbital I). Cryst Struct Commun.

[76] Craven BM, Vizzini EA, Rodrigues MM (1969). The crystal structure of two polymorphs of 5,5′-diethylbarbituric acid (barbital). Acta Crystallogr Sect B: Struct Sci.

[77] Chentli-Benchikha F, Declercq JP, Germain G, Van Meerssche M, Bouche R, Draguet-Brughmans M (1977). Structures des oxo-3 et oxo-6 cyclobarbitals. Acta Crystallogr Sect B: Struct Sci.

[78] Gelbrich T, Rossi D, Griesser UJ (2012). Tetragonal polymorph of 5,5-dichlorobarbituric acid. Acta Crystallogr Sect E: Struct Rep Online.

[79] Gartland GL, Craven BM (1971). The crystal structure of 5-ethyl-5-(3,3-dimethylbutyl)barbituric acid (γ-methylamobarbital). Acta Crystallogr Sect B: Struct Sci.

[80] Nichol GS, Clegg W (2007). 5-(1-Methylbutyl)-5-propenylbarbituric acid (quinal barbitone). Acta Crystallogr Sect E: Struct Rep Online.

[81] Gatehouse BM, Craven BM (1971). The crystal structures of monoclinic 5-ethylbarbituric acid and 5-hydroxy-5-ethylbarbituric acid. Acta Crystallogr Sect B: Struct Sci.

[82] Bravic G, Housty J, Bideau JP (1968). Crystal structure of methylphenylbarbital (mephebarbital). C R Acad Sci, Ser C: Sci Chim.

[83] Gelbrich T, Zencirci N, Griesser UJ (2007). A polymorph of butobarbital with two distinct hydrogen-bonding motifs. Acta Crystallogr Sect C: Cryst Struct Commun.

[84] Craven BM, Vizzini EA (1971). The crystal structure of polymorph IV of 5,5-diethylbarbituric acid (barbital). Acta Crystallogr Sect B: Struct Sci.

[85] Craven BM, Cusatis C (1969). The crystal structure of 5-ethyl-5-(1-methylbutenyl)-barbituric acid. Acta Crystallogr Sect B: Struct Sci.

