# Supplementary material for: Specific energy contributions from competing hydrogen-bonded structures in six polymorphs of phenobarbital
Source: Chem Cent J. 2016 Feb 22;10:8. doi: 10.1186/s13065-016-0152-5 (PMC4763432; doi:10.1186/s13065-016-0152-5)
Supplement: Supplementary file 1 — 10.1186/s13065-016-0152-5 Details of the preparation of forms V and VI, thermal ellipsoid plots for V and VI, structural information about forms I–III, IR spectra of V and VI, details of PIXEL calculations and XPac comparisons, derived structure model for X. [file 13065_2016_152_MOESM1_ESM.pdf]

Supporting Information

Specific energy contributions from competing hydrogen-bonded structures in six polymorphs of phenobarbital

Thomas Gelbrich,* **Doris E. Braun and Ulrich Griesser**

Institute of Pharmacy, University of Innsbruck, Innrain 52, 6020 Innsbruck, Austria.

thomas.gelbrich@uibk.ac.at

Contents

1. Preparation of polymorphs **V** and **VI** 3
2. Thermal ellipsoid plots for forms **V** and **VI** 4
3. Structural features of forms **I**, **II** and **III** 5
4. IR spectroscopy 7
   4.1. Experimental 7
   4.2. Results 7
5. PIXEL calculations 8
   5.1. Overview 8
   5.2. Form **I** 9
   5.3. Form **II** 10
   5.4. Form **III** 12
   5.5. Form **V** 12
   5.6. Form **VI** 13
   5.7. Form **X** 14
6. Intramolecular energy penalties 15
7. *XPac* comparisons with theoretical structures 16
   7.1. Method 16
   7.2. Form **V** 16
   7.3. Form **X** 17
8. Structure model for polymorph **X** 18
9. References 19

1. Preparation of polymorphs V and VI

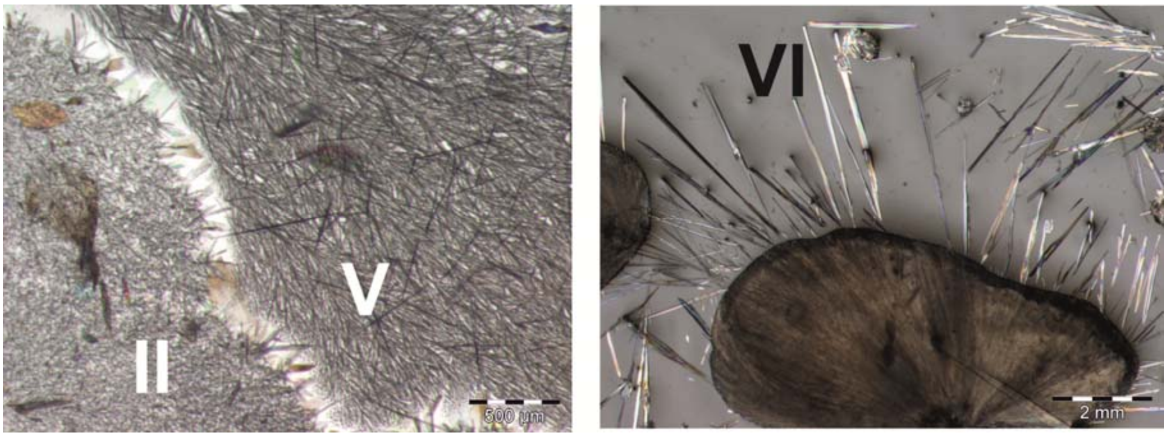

Fig. S1. Left: Pbtl forms **II** and **V** produce by sublimation at 135 °C. The two phases are separated by a transformation frontier (scale bar = 500 μm). Right: Polycrystalline aggregate and prismatic single crystals of polymorph**VI** in paraffin oil after crystallization at 100 °C (scale bar = 2 mm).

2. Thermal ellipsoid plots for forms V and VI

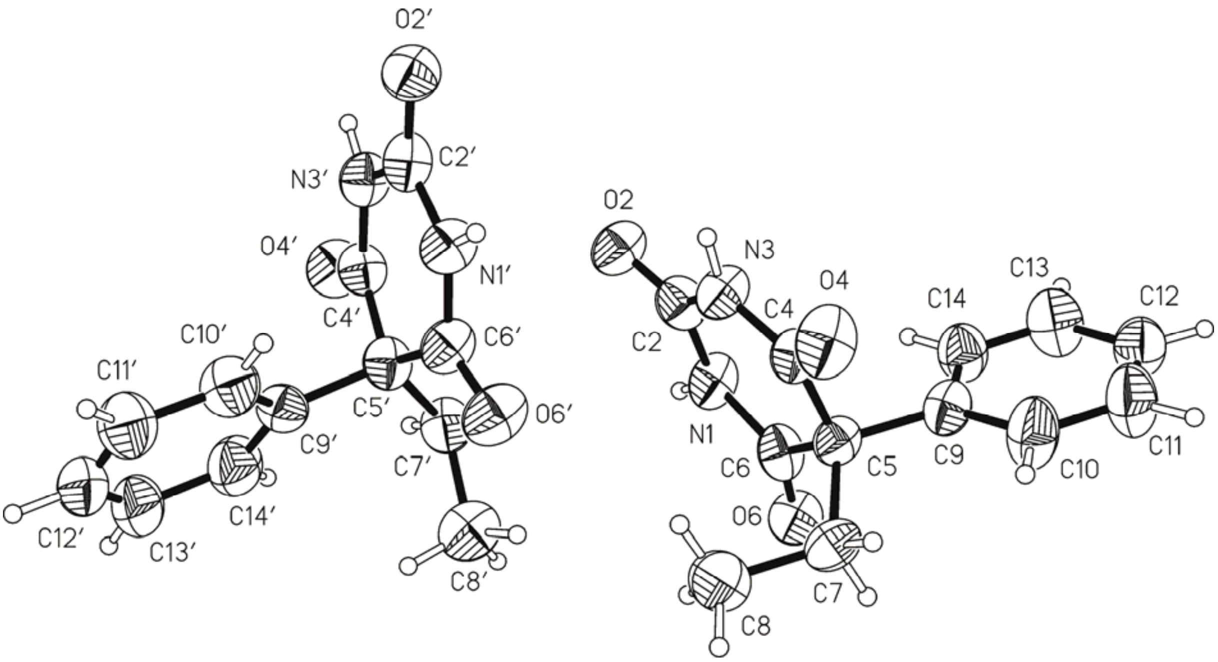

Fig. S2. Asymmetric unit of form **V** of Pbtl with thermal ellipsoids drawn at the 50% probability level.

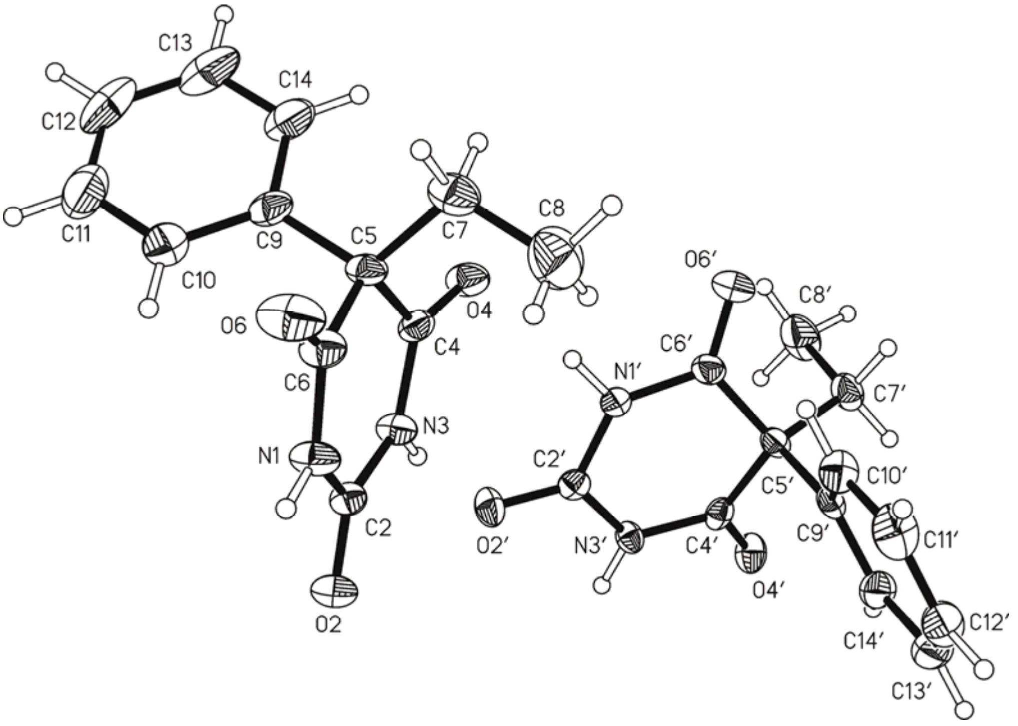

Fig. S3. Asymmetric unit of form **VI** of Pbtl with thermal ellipsoids drawn at the 50% probability level.

3. Structural features of forms I, II and III

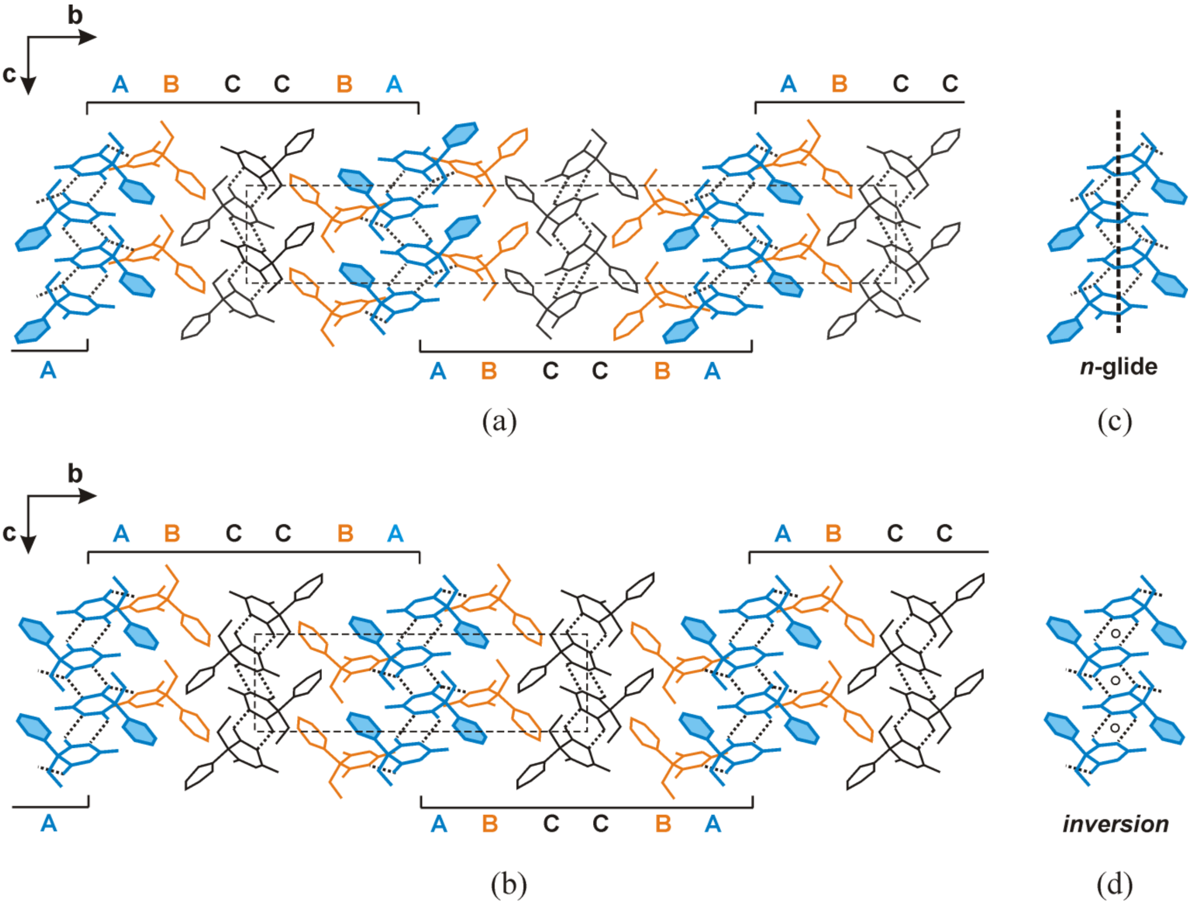

Fig. S4. Illustration of the close packing relationship between polymorphs **I** (**a**) and **II** (**b**) of Pbtl, both containing three symmetry-independent molecules, labelled A – C. In each structure, BAAB sequences correspond with a N–H···O bonded **L-3** layer topology and the CC sequences with a **C-2** chain (see Fig. 1). The only fundamental difference between the two forms is the symmetry of **C-2** chains composed of C-type molecules, which in form **I** is a glide symmetry (**c**) and an inversion symmetry in form **II** (**d**). For more details, see ref. [1].

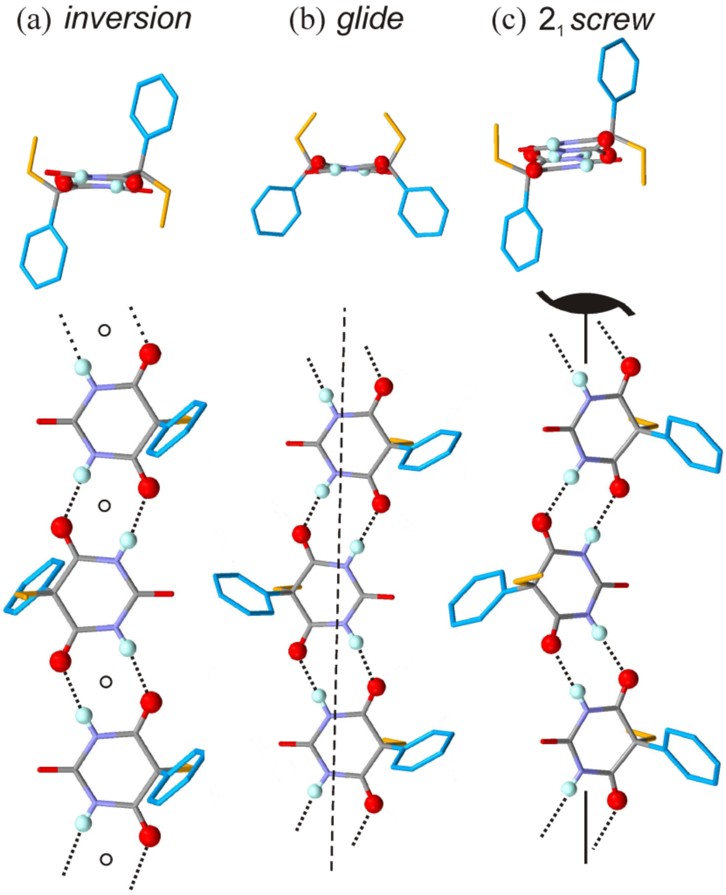

Fig. S5. **C-2** chains of different symmetry in polymorphs of Pbtl: (**a**) inversion (**I**: molecule B; **II**: molecules B and C); (**b**) glide plane (**I**: molecule C); (**c**) 2_1 axis (**III**). Each **C-2** chain is viewed parallel (top) and perpendicular to its direction of translation.

4. IR spectroscopy

4.1. Experimental

FT-IR spectra were recorded with a Bruker IFS 25 spectrometer connected with the IR microscope I (Bruker). The samples were prepared on ZnSe discs and measured in transmission mode (15x Cassegrain objective, spectral range 4000 to 600 cm^{-1}, resolution 4 cm^{-1}, 64 interferograms per spectrum).

4.2. Results

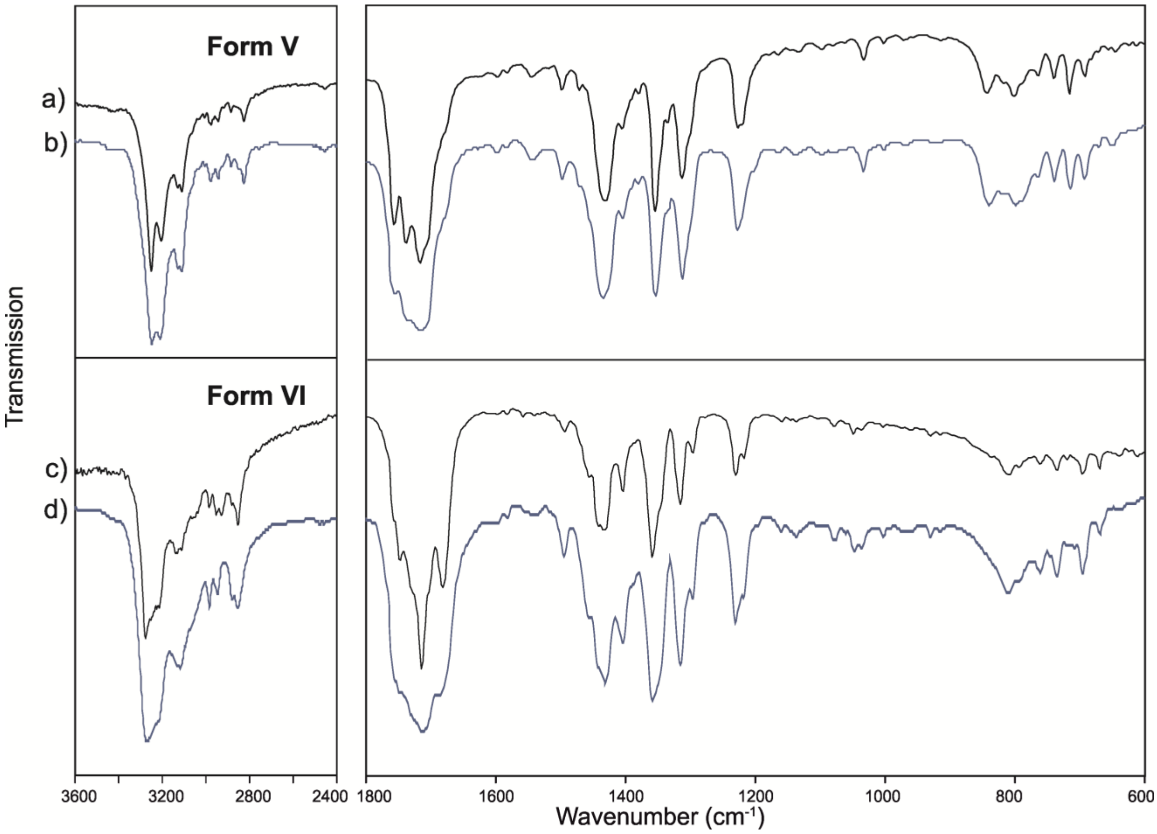

Fig. S6. Comparison of IR spectra for polymorphs **V** (a) and **VI** (c) of Pbtl obtained of in this study (a, c) with reference spectra (b, d) recorded in a previous study [1].

5. PIXEL calculations

5.1. Overview

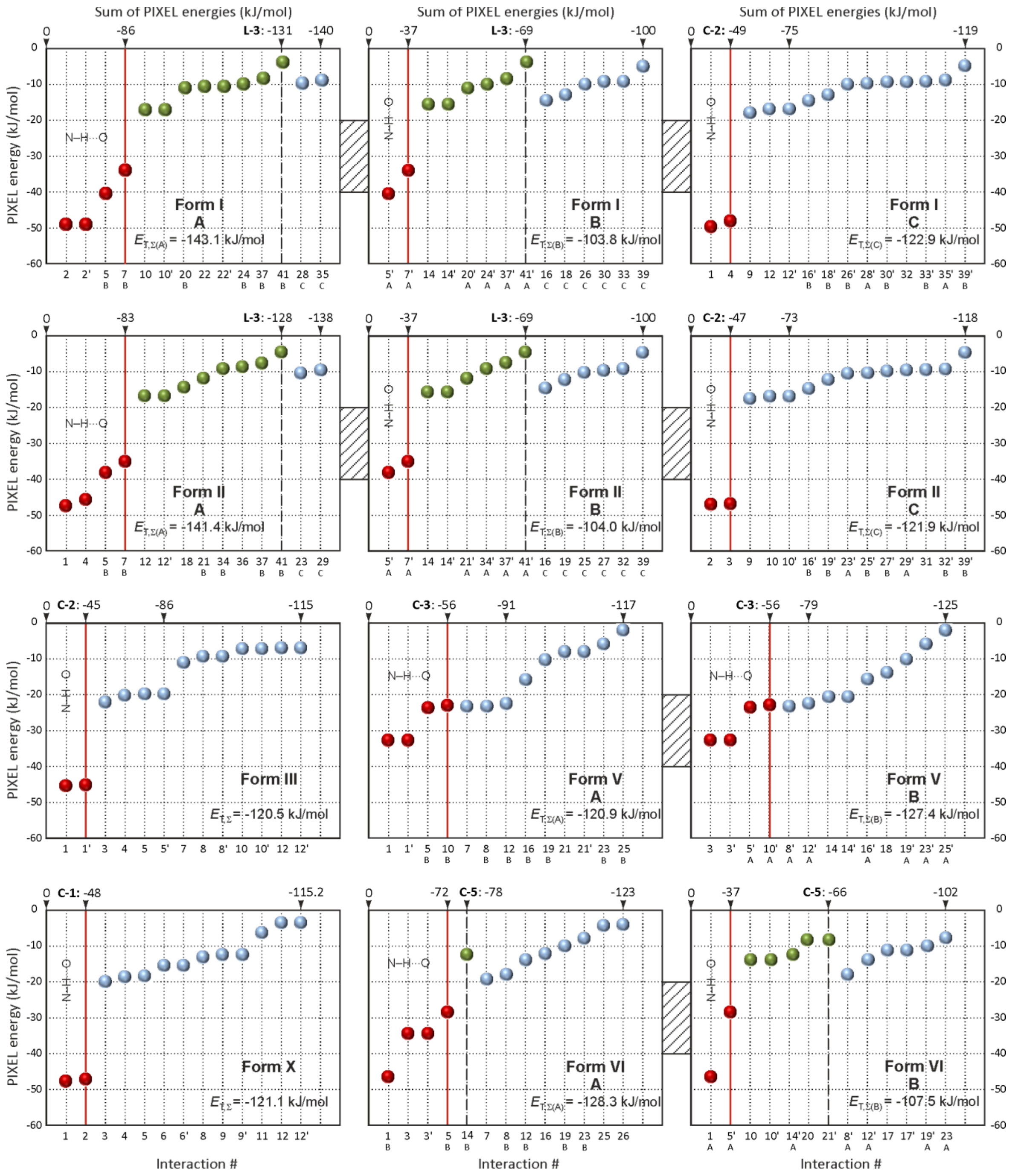

Fig. S7. PIXEL interaction energies for polymorphs of Pbtl. Red balls indicate H-bond interactions and green balls non-H bond interactions within the same instance of an HBS. All other interactions are represented by grey balls.

5.2. Form I

$E_{T,\Sigma}$ = –123.3 kJ mol^{-1} ($E_{T,Cry}$ not calculated)
$E_{T,\Sigma(A)}$ = –143.1 kJ mol^{-1}
$E_{T,\Sigma(B)}$ = –103.8 kJ mol^{-1}
$E_{T,\Sigma(C)}$ = –122.9 kJ mol^{-1}

Table S1. Pairwise interaction energies (kJ mol^{-1}) for polymorph **I** of Pbtl (molecule A).

#	Type	Mol.	Symmetry operation	*d* (Å)	E_C	E_P	E_D	E_R	E_T
2	**L-3_t**	A	–1/2+*x*, 1/2–*y*, –1/2+z	7.019	–60.2	–22.1	–19.8	52.8	–49.2
2'	**L-3_t**	A	1/2+*x*, 1/2–*y*, 1/2+z	7.019	–60.2	–22.1	–19.8	52.8	–49.2
5	**L-3_o**	B	*x*, *y*, *z*	6.352	–27.3	–8.9	–29.3	24.9	–40.5
7	**L-3_o**	B	*x*–1, *y*, z	6.806	–31.0	–9.9	–23.4	30.3	–34.0
10	**L-3_n**	A	*x* , *y*, z–1	6.803	–5.9	–2.7	–20.8	12.1	–17.2
10'	**L-3_n**	A	*x*, *y*, z+1	6.803	–5.9	–2.7	–20.8	12.1	–17.2
20	**L-3_n**	B	–1/2+*x*, 1/2–*y*, –1/2+z	7.794	–3.8	–2.8	–13.1	8.6	–11.1
22	**L-3_n**	A	–1/2+*x*, 1/2–*y*, 1/2+z	7.389	–1.2	–1.1	–11.2	2.8	–10.7
22'	**L-3_n**	A	1/2+*x*, 1/2–*y*, –1/2+z	7.389	–1.2	–1.1	–11.2	2.8	–10.7
24	**L-3_n**	B	–1/2+*x*, 1/2–*y*, 1/2+z	8.208	–2.3	–2.1	–9.8	4.3	–10.0
28	**@1a**	C	*x*, *y*, *z*	9.011	–2.3	–1.0	–12.5	6.1	–9.7
35	**@1c**	C	*x*, *y*, z+1	9.185	–1.2	–0.8	–10.9	4.1	–8.9
37	**L-3_n**	B	*x* , *y*, z–1	9.238	–6.3	–2.5	–9.4	9.8	–8.4
41	**L-3_n**	B	*x*–1, *y*, z–1	8.980	–0.6	–0.1	–3.3	0.2	–3.8

Table S2. Pairwise interaction energies (kJ mol^{-1}) for polymorph **I** of Pbtl (molecule B).

#	Type	Mol.	Symmetry operation	*d* (Å)	E_C	E_P	E_D	E_R	E_T
5'	**L-3_o**	A	*x*, *y*, *z*	6.352	–27.3	–8.9	–29.3	24.9	–40.5
7'	**L-3_o**	A	*x*+1, *y*, z	6.806	–31.0	–9.9	–23.4	30.3	–34.0
14	**L-3_n**	B	*x*, *y*, z–1	6.803	–3.7	–1.6	–18.8	8.6	–15.5
14'	**L-3_n**	B	*x*, *y*, z+1	6.803	–3.7	–1.6	–18.8	8.6	–15.5
16	**@1a**	C	*x*+1, *y*, *z*+1	7.459	–7.4	–3.5	–18.6	15.1	–14.5
18	**@1b**	C	*x*+1, *y*, *z*	8.027	–3.4	–1.5	–14.8	6.8	–12.9
20'	**L-3_n**	A	1/2+*x*, 1/2–*y*, 1/2+*z*	7.794	–3.8	–2.8	–13.1	8.6	–11.1
24'	**L-3_n**	A	1/2+*x*, 1/2–*y*, –1/2+z	8.208	–2.3	–2.1	–9.8	4.2	–10.0
26	**@1c**	C	*x*, *y*, z+1	8.019	–0.4	–1.6	–13.5	5.5	–10.0
30	**@1a**	C	*x*, *y*, *z*	7.902	–0.4	–1.8	–15.5	8.4	–9.3
33	**@1a**	C	1–*x*, 1–*y*, 1–*z*	7.993	1.0	–1.0	–12.3	3.2	–9.2
37'	**L-3_n**	A	*x*, *y*, z+1	9.238	–6.3	–2.5	–9.4	9.8	–8.4
39	**@1b**	C	1–*x*, 1–*y*, –*z*	10.293	–3.4	–1.5	–5.2	5.3	–4.9
41'	**L-3_n**	A	*x*+1, *y*, *z*+1	8.980	–0.6	–0.1	–3.3	0.2	–3.8

Table S3. Pairwise interaction energies (kJ mol^{-1}) for polymorph **I** of Pbtl (molecule C).

#	Type	Mol.	Symmetry operation	*d* (Å)	E_C	E_P	E_D	E_R	E_T
1	**C-2**	C	–*x*, 1–*y*, –*z*	6.888	–64.4	–24.2	–20.6	59.4	–49.7
4	**C-2**	C	1–*x*, 1–*y*, 1–*z*	7.125	–67.2	–25.5	–20.7	65.3	–48.1
9	**@2**	C	1–*x*, 1–*y*, –*z*	6.958	–9.5	–3.8	–15.0	10.4	–18.0
12	**@2**	C	*x*, *y*, *z*–1	6.803	–4.3	–1.7	–21.0	10.0	–16.9
12'	**@3**	C	*x*, *y*, *z*+1	6.803	–4.3	–1.7	–21.0	10.0	–16.9
16'	**@1**	B	*x*–1, *y*, *z*–1	7.459	–7.4	–3.5	–18.6	15.1	–14.5
18'	**@1**	B	*x*–1, *y*, *z*	8.027	–3.4	–1.5	–14.8	6.8	–12.9
26'	**@1**	B	*x*, *y*, *z*–1	8.019	–0.4	–1.6	–13.5	5.5	–10.0
28'	**@1**	A	*x*, *y*, *z*	9.011	–2.3	–1.0	–12.5	6.1	–9.7
30'	**@1**	B	*x*, *y*, *z*	7.902	–0.4	–1.8	–15.5	8.4	–9.3
32	**@3**	C	–*x*, 1–*y*, 1–*z*	7.776	–1.1	–1.4	–14.0	7.2	–9.3
33'	**@1**	B	1–*x*, 1–*y*, 1–*z*	7.993	1.0	–1.0	–12.3	3.2	–9.2
35'	**@1**	A	*x*, *y*, *z*–1	9.185	–1.2	–0.8	–10.9	4.1	–8.9
39'	**@1**	B	1–*x*, 1–*y*, –*z*	10.293	–3.4	–1.5	–5.2	5.3	–4.9

#9: Two symmetry-related contacts (phenyl)C33–H33···O7 (H···O 2.65 Å, CHO = 134°)

5.3. Form II

$E_{T,\Sigma}$ = –122.4 kJ mol^{-1} ($E_{T,Cry}$ not calculated)
$E_{T,\Sigma(A)}$ = –141.4 kJ mol^{-1}
$E_{T,\Sigma(B)}$ = –104.0 kJ mol^{-1}
$E_{T,\Sigma(C)}$ = –121.9 kJ mol^{-1}

Table S4. Pairwise interaction energies (kJ mol^{-1}) for polymorph **II** of Pbtl (molecule A).

#	Type	Mol.	Symmetry operation	*d* (Å)	E_C	E_P	E_D	E_R	E_T
1	**L-3_t**	A	–*x*+1, –*y*+1, –*z*+2	6.968	–61 .9	–23 .1	–19 .8	57 .3	–47 .5
4	**L-3_t**	A	–*x*, –*y*+1, –*z*+1	7.118	–61 .6	–23 .6	–20 .8	60 .2	–45 .7
5	**L-3_o**	B	*x*–1, *y*, *z*	6.326	–25 .9	–9 .4	–30 .2	27 .4	–38 .2
7	**L-3_o**	B	*x*, *y*, *z*	6.788	–35 .1	–11 .6	–24 .6	36 .2	–35 .1
12	**L-3_n**	A	*x*, *y*, *z*–1	6.719	–5 .9	–2 .6	–22 .3	13 .9	–16 .8
12’	**L-3_n**	A	*x*, *y*, *z*+1	6.719	–5 .9	–2 .6	–22 .3	13 .9	–16 .8
18	**L-3_n**	A	–*x*, –*y*+1, –*z*+2	6.860	–6 .1	–3 .1	–13 .6	8 .2	–14 .4
21	**L-3_n**	B	–*x*+1, –*y*+1, –*z*+1	7.914	–4 .2	–3 .1	–15 .7	11 .2	–11 .9
34	**L-3_n**	B	*x*–1, *y*, *z*+1	9.066	–6 .4	–2 .5	–10 .3	10 .0	–9 .2
36	**L-3_n**	A	–*x*+1, –*y*+1, –*z*+1	7.967	–0 .2	–0 .6	–10 .5	2 .8	–8 .6
37	**L-3_n**	B	–*x*+1, –*y*+1, –*z*+2	7.874	–1 .6	–1 .9	–9 .0	5 .0	–7 .6
41	**L-3_n**	B	*x*, *y*, *z*+1	8.771	–0 .5	–0 .2	–4 .4	0 .5	–4 .6
23	**@1b**	C	*x*, *y*, *z*+1	8.880	–3 .3	–1 .6	–15 .0	9 .3	–10 .5
29	**@1a**	C	*x*, *y*, *z*	9.072	–1 .8	–0 .9	–11 .9	5 .0	–9 .6

Table S5. Pairwise interaction energies (kJ mol^{-1}) for polymorph **II** of Pbtl (molecule B).

#	Type	Mol.	Symmetry operation	*d* (Å)	E_C	E_P	E_D	E_R	E_T
5’	**L-3_o**	A	*x*+1, *y*, *z*	6.326	−25 .9	−9 .4	−30 .2	27 .4	−38 .2
7’	**L-3_o**	A	*x*, *y*, *z*	6.788	−35 .1	−11 .6	−24 .6	36 .2	−35 .1
14	**L-3_n**	B	*x*, *y*, *z*−1	6.719	−4 .7	−2 .1	−20 .2	11 .3	−15 .7
14’	**L-3_n**	B	*x*, *y*, *z*+1	6.719	−4 .7	−2 .1	−20 .2	11 .3	−15 .7
21’	**L-3_n**	A	−*x*+1, −*y*+1, −*z*+1	7.914	−4 .2	−3 .1	−15 .7	11 .2	−11 .9
34’	**L-3_n**	A	*x*+1, *y*, *z*−1	9.066	−6 .4	−2 .5	−10 .3	10 .0	−9 .2
37’	**L-3_n**	A	−*x*+1 −*y*+1, −*z*+2	7.874	−1 .6	−1 .9	−9 .0	5 .0	−7 .6
41’	**L-3_n**	A	*x*, *y*, *z*−1	8.771	−0 .5	−0 .2	−4 .4	0 .5	−4 .6
16	**@1b**	C	*x*, *y*, *z*	7.401	−9 .1	−4 .3	−19 .4	18 .1	−14 .7
19	**@1a**	C	*x*, *y*, *z*+1	8.101	−3 .3	−1 .5	−14 .6	7 .0	−12 .3
25	**@1a**	C	−*x*+1, −*y*+2, −*z*+1	7.939	0 .3	−1 .4	−14 .0	4 .8	−10 .3
27	**@1c**	C	*x*+1, *y*, *z*	7.932	−0 .3	−1 .9	−15 .1	7 .5	−9 .8
32	**@1a**	C	*x*+1, *y*, *z*+1	7.903	−0 .6	−2 .0	−16 .1	9 .3	−9 .3
39	**@1b**	C	−*x*+1, −*y*+2, −*z*+2	10.283	−3 .7	−1 .6	−5 .4	6 .1	−4 .7

#16: Contact (phenyl)C35–H35···O5 (H···O 2.40 Å, CHO = 163°)

Table S6. Pairwise interaction energies (kJ mol^{-1}) for polymorph **II** of Pbtl (molecule C).

#	Type	Mol.	Symmetry operation	*d* (Å)	E_C	E_P	E_D	E_R	E_T
2	**C-2**	C	−*x*, −*y*+2, −*z*	7.093	−71 .1	−27 .3	−21 .4	72 .8	−47 .0
3	**C-2**	C	−*x*+1, −*y*+2, −*z*+1	6.864	−67 .3	−25 .5	−21 .2	67 .2	−46 .8
9	**@2**	C	−*x*, −*y*+2, −*z*+1	6.953	−9 .8	−3 .8	−15 .4	11 .3	−17 .6
10	**@3**	C	*x*, *y*, *z*−1	6.719	−5 .3	−2 .2	−23 .4	14 .0	−16 .9
10’	**@2**	C	*x*, *y*, *z*+1	6.719	−5 .3	−2 .2	−23 .4	14 .0	−16 .9
16’	**@1**	B	*x*, *y*, *z*	7.401	−9 .1	−4 .3	−19 .4	18 .1	−14 .7
19’	**@1**	B	*x*, *y*, *z*−1	8.101	−3 .3	−1 .5	−14 .6	7 .0	−12 .3
23’	**@1**	A	*x*, *y*, *z*−1	8.880	−3 .3	−1 .6	−15 .0	9 .3	−10 .5
25’	**@1**	B	−*x*+1, −*y*+2, −*z*+1	7.939	0 .3	−1 .4	−14 .0	4 .8	−10 .3
27’	**@1**	B	*x*−1, *y*, *z*	7.932	−0 .3	−1 .9	−15 .1	7 .5	−9 .8
29’	**@1**	A	*x*, *y*, *z*	9.072	−1 .8	−0 .9	−11 .9	5 .0	−9 .6
31	**@3**	C	−*x*+1, −*y*+2, −*z*	7.771	−1 .3	−1 .5	−14 .4	7 .8	−9 .5
32’	**@1**	B	*x*−1, *y*, *z*−1	7.903	−0 .6	−2 .0	−16 .1	9 .3	−9 .3
39’	**@1**	B	−*x*+1, −*y*+1, −*z*+2	10.283	−3 .7	−1 .6	−5 .4	6 .1	−4 .7

#9: Two symmetry-related contacts (phenyl)C33–H33···O7 (H···O 2.62 Å, CHO = 134°)
#16’: Contact (phenyl)C35–H35···O5 (H···O 2.40 Å, CHO = 163°)

5.4. Form III

$E_{T,Cry}$ = −118.3 kJ mol^{-1}
$E_{T,\Sigma}$ = −120.5 kJ mol^{-1}

Table S7. Pairwise interaction energies (kJ mol^{-1}) for polymorph **III** of Pbtl.

#	Type	Symmetry operation	*d* (Å)	E_C	E_P	E_D	E_R	E_T
1	**C-2**	2−*x*, −1/2+*y*, 1/2−*z*	7.052	−60.3	−23.8	−21.0	59.7	−45.4
1’	**C-2**	2−*x*, 1/2+*y*, 1/2−*z*	7.052	−60.3	−23.8	−21.0	59.7	−45.3
3	**@3**	1−*x*, −*y*, −*z*	6.183	−3.9	−2.5	−30.7	15.1	−22.1
4	**@1**	2−*x*, −*y*, 1−*z*	6.807	−13.2	−4.9	−17.3	15.1	−20.2
5	**@1**	*x*, 1/2−*y*, 1/2+*z*	6.313	−4.4	−2.8	−27.0	14.5	−19.7
5’	**@2**	*x*, 1/2−*y*, −1/2+*z*	6.313	−4.4	−2.8	−27.0	14.5	−19.7
7	**@2**	2−*x*, −*y*, −*z*	7.450	−5.1	−2.7	−22.8	19.6	−11.0
8	**@4**	1−*x*, −1/2+*y*, 1/2−*z*	8.393	−1.5	−1.0	−9.1	2.4	−9.2
8’	**@4**	1−*x*, 1/2+*y*, 1/2−*z*	8.393	−1.5	−1.0	−9.1	2.4	−9.2
10	**@3**	*x*−1, 1/2−*y*, −1/2+*z*	9.643	−5.5	−1.5	−5.0	4.9	−7.1
10’	**@5**	*x*+1, 1/2−*y*, 1/2+*z*	9.643	−5.5	−1.5	−5.0	4.9	−7.2
12	**@4**	*x*−1, *y*, *z*	9.546	−1.7	−1.1	−7.9	3.9	−6.8
12’	**@6**	*x*+1, *y*, *z*	9.546	−1.7	−1.1	−7.9	3.9	−6.8

#4: Two symmetry-related contacts (phenyl)C12–H12···O1 (H···O 2.58 Å, CHO = 143°)
#10/10’: Two symmetry-related contacts (phenyl)C9–H9···O1 (H···O 2.61 Å, CHO = 151°)

5.5. Form V

$E_{T,Cry}$ = −122.2 kJ mol^{-1}
$E_{T,\Sigma}$ = −124.1 kJ mol^{-1}
$E_{T,\Sigma(A)}$ = −120.9 kJ mol^{-1}
$E_{T,\Sigma(B)}$ = −127.4 kJ mol^{-1}

Table S8. Pairwise interaction energies (kJ mol^{-1}) for polymorph **V** (molecule A).

#	Type	Mol.	Symmetry operation	*d* (Å)	E_C	E_P	E_D	E_R	E_T
1	**C-3**	A	*x*, *y*−1, *z*	6.762	−41.8	−16.9	−19.4	45.1	−32.9
1’	**C-3**	A	*x*, *y*+1, *z*	6.762	−41.8	−16.9	−19.4	45.1	−32.9
5	**C-3**	B	−*x*, −*y*, −*z*	7.969	−36.9	−15.4	−11.0	39.6	−23.8
7	**@1**	A	2−*x*, 1−*y*, 2−*z*	6.222	−4.4	−3.7	−30.7	15.3	−23.5
8	**@1**	B	*x*+1, *y*, *z*	6.815	−6.8	−3.1	−23.4	9.9	−23.5
10	**C-3**	B	1−*x*, 2−*y*, 2−*z*	8.172	−29.9	−10.6	−9.5	26.9	−23.2
12	**@2**	B	*x*, *y*, *z*	6.812	−11.7	−5.6	−28.4	23.0	−22.7
16	**@1**	B	*x*+1, *y*−1, *z*	7.784	−4.7	−2.4	−21.9	13.0	−16.0
19	**@2**	B	*x*, *y*−1, *z*	7.781	−4.0	−1.2	−11.0	5.7	−10.5
21	**@4**	A	3/2−*x*, −1/2+*y*, 3/2−*z*	8.527	−4.0	−1.7	−10.8	8.3	−8.3
21’	**@4**	A	3/2−*x*, 1/2+*y*, 3/2−*z*	8.527	−4.0	−1.7	−10.8	8.3	−8.3
23	**@5**	B	3/2−*x*, −1/2+*y*, 3/2−*z*	8.463	−2.2	−0.8	−8.0	4.8	−6.2
25	**@6**	B	1/2−*x*, 1/2+*y*, 3/2−*z*	10.931	0.3	−0.2	−3.5	1.0	−2.4

#12/12': Contact (CH_2)C7'–H7'1···O2 (H···O 2.58 Å, CHO = 143°)

Table S9. Pairwise interaction energies (kJ mol^{-1}) for polymorph **V** (molecule B).

#	Type	Mol.	Symmetry operation	*d* (Å)	E_C	E_P	E_D	E_R	E_T
3	**C-3**	B	*x*, *y*–1, *z*	6.762	–35.6	–13.5	–17.4	33.5	–32.9
3'	**C-3**	B	*x*, *y*+1, *z*	6.762	–35.6	–13.5	–17.4	33.5	–32.9
5'	**C-3**	A	1–*x*, 1–*y*, 2–*z*	7.969	–36.9	–15.4	–11.0	39.6	–23.7
8'	**@1**	A	*x*–1, *y*, *z*	6.815	–6.8	–3.1	–23.4	9.9	–23.5
10'	**C-3**	A	1–*x*, 2–*y*, 2–*z*	8.172	–29.9	–10.6	–9.5	26.9	–23.1
12'	**@2**	A	*x*, *y*, *z*	6.812	–11.7	–5.6	–28.4	23.0	–22.7
14	**@3**	B	1/2–*x*, –1/2+*y*, 3/2–*z*	7.628	–5.7	–2.4	–24.9	12.0	–20.9
14'	**@3**	B	1/2–*x*, 1/2+*y*, 3/2–*z*	7.628	–5.7	–2.4	–24.9	12.0	–20.9
16'	**@1**	A	*x*–1, *y*+1, *z*	7.784	–4.7	–2.4	–21.9	13.0	–16.0
18	**@2**	B	1–*x*, 1–*y*, 2–*z*	8.982	–9.0	–1.8	–7.8	4.5	–14.1
19'	**@2**	A	*x*, *y*+1, *z*	7.781	–4.0	–1.2	–11.0	5.7	–10.5
23'	**@5**	A	3/2–*x*, 1/2+*y*, 3/2–*z*	8.463	–2.2	–0.8	–8.0	4.8	–6.2
25'	**@6**	A	1/2–*x*, 1/2+*y*, 3/2–*z*	10.931	0.3	–0.2	–3.5	1.0	–2.4

#12, #12': Single contact (CH_2)C7'–H7'1···O2 (H···O 2.58 Å, CHO = 143°)

5.6. Form VI

$E_{T,Cry}$ = –114.9 kJ mol^{-1}
$E_{T,\Sigma}$ = –117.9 kJ mol^{-1}
$E_{T,\Sigma(A)}$ = –128.3 kJ mol^{-1}
$E_{T,\Sigma(B)}$ = –107.5 kJ mol^{-1}

Table S10. Pairwise interaction energies (kJ mol^{-1}) for polymorph **VI** (molecule A).

#	Type	Mol.	Symmetry operation	d (Å)	E_C	E_P	E_D	E_R	E_T
1	**L-5_t**	B	*x*, *y*, *z*	7.071	–63.7	–25.1	–22.6	64.9	–46.5
3	**L-5_o**	A	*x*, *y*–1, *z*	6.900	–27.2	–10.2	–20.7	23.7	–34.4
3'	**L-5_o**	A	*x*, *y*+1, *z*	6.900	–27.2	–10.2	–20.7	23.7	–34.4
5	**L-5_o**	B	3/2–*x*, 1/2+*y*, 1/2–*z*	7.028	–36.0	–15.0	–16.8	39.4	–28.4
7	**@1**	A	1–*x*, 1–*y*, –*z*	6.717	–3.2	–2.6	–24.5	11.2	–19.2
8	**@1**	B	–1/2+*x*, 1/2–*y*, –1/2+z	6.948	–4.2	–2.4	–23.0	11.6	–17.9
12	**@1**	B	–1/2+*x*, –1/2–*y*, –1/2+*z*	8.419	–6.4	–2.4	–16.4	11.3	–13.9
14	**L-5_n**	B	*x*, *y*+1, *z*	7.750	–6.2	–2.6	–12.8	9.1	–12.4
16	**@1**	A	1–*x*, –*y*, –*z*	7.593	–0.2	–0.7	–12.8	1.6	–12.1
19	**@2**	B	2–*x*, –*y*, –*z*	7.394	–1.3	–1.4	–13.6	6.3	–10.0
23	**@2**	B	2–*x*, 1–*y*, –*z*	8.926	–5.1	–1.2	–4.7	3.1	–7.8
25	**@2**	A	2–*x*, –*y*, –*z*	9.513	–0.6	–0.1	–4.3	0.7	–4.3
26	**@2**	A	2–*x*, 1–*y*, –*z*	8.829	–0.7	–0.8	–7.2	4.8	–4.0

Table S11. Pairwise interaction energies (kJ mol^{-1}) for polymorph **VI** (molecule B).

#	Type	Mol.	Symmetry operation	*d* (Å)	E_C	E_P	E_D	E_R	E_T
1’	**L-5_t**	A	*x*, *y*, *z*	7.071	–63.7	–25.1	–22.6	64.9	–46.5
5’	**L-5_o**	A	3/2–*x*, –1/2+*y*, 1/2–*z*	7.028	–36.0	–15.0	–16.8	39.4	–28.4
8’	**@1**	A	1/2+*x*, 1/2–*y*, 1/2+z	6.948	–4.2	–2.4	–23.0	11.6	–17.9
10	**L-5_n**	B	*x*, *y*–1, *z*	6.900	–7.5	–3.1	–26.3	22.9	–13.9
10’	**L-5_n**	B	*x*, *y*+1, *z*	6.900	–7.5	–3.1	–26.3	22.9	–13.9
12’	**@1**	A	1/2+*x*, –1/2–*y*, 1/2+z	8.419	–6.4	–2.4	–16.4	11.3	–13.9
14’	**L-5_n**	A	*x*, *y*–1, *z*	7.750	–6.2	–2.6	–12.8	9.1	–12.4
17	**@3**	B	5/2–*x*, –1/2+*y*, 1/2–*z*	8.569	–6.8	–3.6	–20.6	19.9	–11.1
17’	**@3**	B	1/2–*x*, 1/2+*y*, 1/2–*z*	8.569	–6.8	–3.6	–20.6	19.9	–11.1
19’	**@2**	A	2–*x*, –*y*, –*z*	7.394	–1.3	–1.4	–13.6	6.3	–10.0
20	**L-5_n**	B	3/2–*x*, –1/2+*y*, 1/2–*z*	7.711	–5.6	–3.0	–12.2	12.4	–8.3
21’	**L-5_n**	B	3/2–*x*, 1/2+*y*, 1/2–*z*	7.711	–5.6	–3.0	–12.2	12.4	–8.3
23’	**@2**	A	2–*x*, 1–*y*, –*z*	8.926	–5.1	–1.2	–4.7	3.1	–7.8

5.7. Form X

$E_{T,Cry}$ = –118.3 kJ mol^{-1}
$E_{T,\Sigma}$ = –121.1 kJ mol^{-1}

Table S12. Pairwise interaction energies (kJ mol^{-1}) for polymorph **X**.

#	Type	Symmetry operation	*d* (Å)	E_C	E_P	E_D	E_R	E_T
1	**C-1**	2–*x*, *y*, 1/2–*z*	6.272	–68.7	–28.9	–22.9	72.8	–47.7
2	**C-1**	2–*x*, *y*, 3/2–*z*	8.001	–67.4	–24.6	–19.2	64.0	–47.2
3	**@1**	3/2–*x*, 1/2–*y*, 1–*z*	6.675	–9.8	–2.9	–15.1	8.0	–19.7
4	**@1**	3/2–*x*, 1/2–*y*, –*z*	7.942	–6.0	–1.8	–18.0	7.5	–18.3
5	**@3**	1–*x*, *y*, 1/2–*z*	6.597	–9.5	–4.1	–19.9	15.4	–18.1
6	**@2**	*x*, 1–y, –1/2+*z*	7.316	–4.1	–1.9	–13.0	3.9	–15.1
6’	**@2**	*x*, 1–y, 1/2+z	7.316	–4.1	–1.9	–13.0	3.9	–15.1
8	**@2**	2–*x*, 1–*y*, 1–*z*	7.256	–9.1	–5.0	–21.4	22.7	–12.8
9	**@1**	–1/2+*x*, 1/2–*y*, –1/2+*z*	7.857	–5.3	–3.4	–20.1	16.7	–12.1
9’	**@4**	1/2+*x*, 1/2–*y*, 1/2+*z*	7.857	–5.3	–3.4	–20.1	16.7	–12.1
11	**@5**	1–*x*, 1–*y*, –*z*	8.680	1.2	–0.7	–10.6	4.2	–5.9
12	**@6**	3/2–x, –1/2+y, 1/2–*z*	10.406	0.6	–0.2	–4.5	0.9	–3.1
12’	**@7**	3/2–x, 1/2+y, 1/2–*z*	10.406	0.6	–0.2	–4.5	0.9	–3.1

#3: Two symmetry-related contacts (phenyl)C9–H9···O3 (H···O 2.68 Å, CHO = 140°)
#5: Two symmetry-related contacts (CH_2)C5–H4···O3 (H···O 2.56 Å, CHO = 155°)
#8: Two symmetry-related contacts (CH_3)C6–H7···O1 (H···O 2.51 Å, CHO = 170°)

6. Intramolecular energy penalties

Table S13. Intramolecular energy penalties (ΔE_{intra}) of the experimental geometries with respect to the global conformational energy minimum, calculated at the MP2/6-31G(d,p) level of theory.

Polymorph	Molecule[a]	ϕ^b / °	ω^b / °	ΔE_{intra} / kJ mol^{-1}
**I**	A	1.1	34.1	8.9
	B	1.1	48.1	6.9
	C	−2.2	44.3	6.0
	crystal	–	–	7.3
**II**	A	0.8	39.5	8.7
	B	2.4	46.2	8.2
	C	−1.0	46.5	5.5
	crystal	–	–	7.5
**III**	A, crystal	−0.4	52.5	3.9
**V**	A	−3.1	30.9	8.5
	**B**	**−129.3**	**31.3**	**17.6**
	crystal	–	–	13.1
**VI**	A	−0.6	76.7	0.3
	B	0.7	42.3	7.1
	crystal	–	–	3.7
**X** (Nbtl·Pbtl)	A, crystal	−6.9	18.3	8.0

[a] Symmetry-independent molecules are denoted by letters (A, B, C) and "crystal" indicates the average ΔE_{intra} value of all geometries in a polymorph. [b]Dihedral angles are defined in Fig. 15.

7. *XPac* comparisons with theoretical structures

7.1. Method

XPac comparison was carried out

7.2. Form V

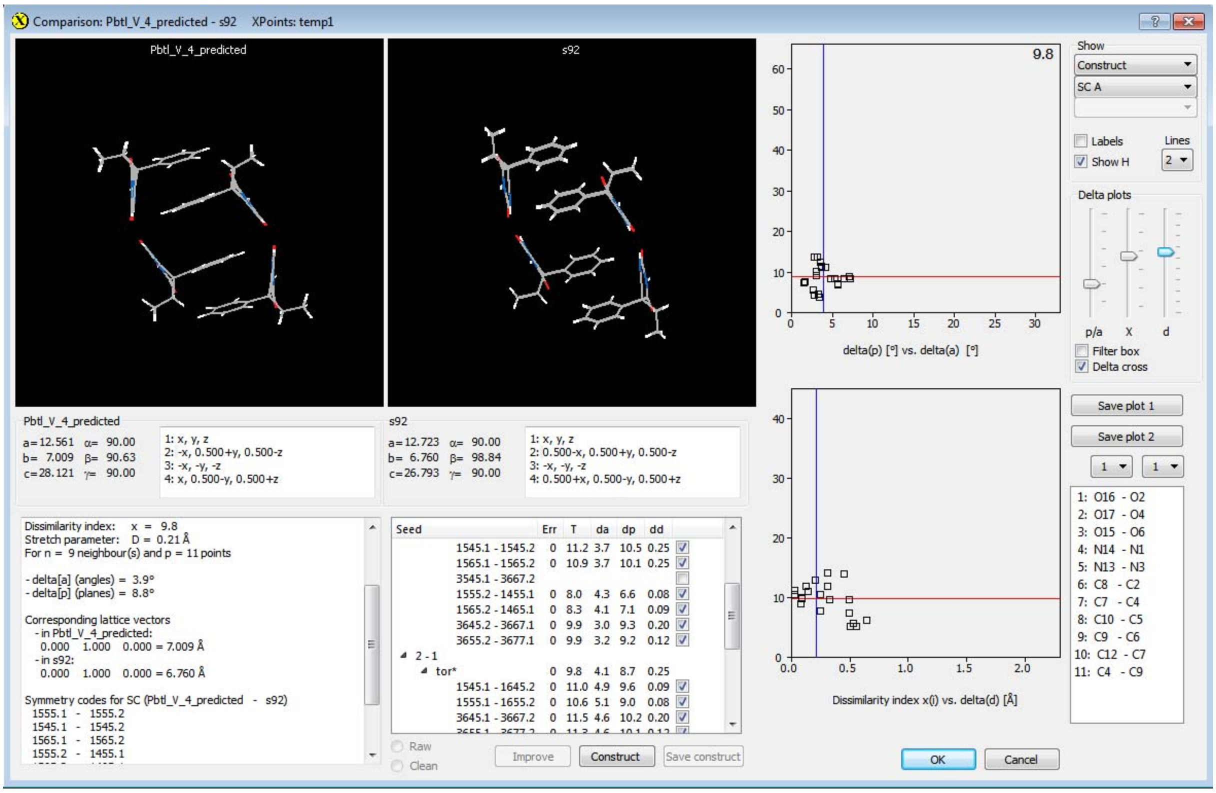

Fig. S8. Screenshot showing a summary of an *XPac* comparison [2] between the experimental crystal structure of form **V** ("s92") and the theoretical candidate structure #4 for form **V** ("Pbtl_V_4_predicted)" reported by Day *et al.* [3] The 11 matching atomic positions listed in the bottom right panel (the complete pyrimidinetrione unit and the first C atom of each of the phenyl and ethyl substituents at ring atom C5) were used to generate geometrical parameters for the *XPac* calculations, which where therefore largely unaffected by any differences in molecular geometry. A stack of centrosymmetric molecule pairs propagating along the respective *b* axis (one-dimensional supramolecular construct) was identified as the closest common structure fragment. The *XPac* dissimilarity index [4] for this common structure fragment of 9.8 indicates significant geometrical differences.

7.3. Form X

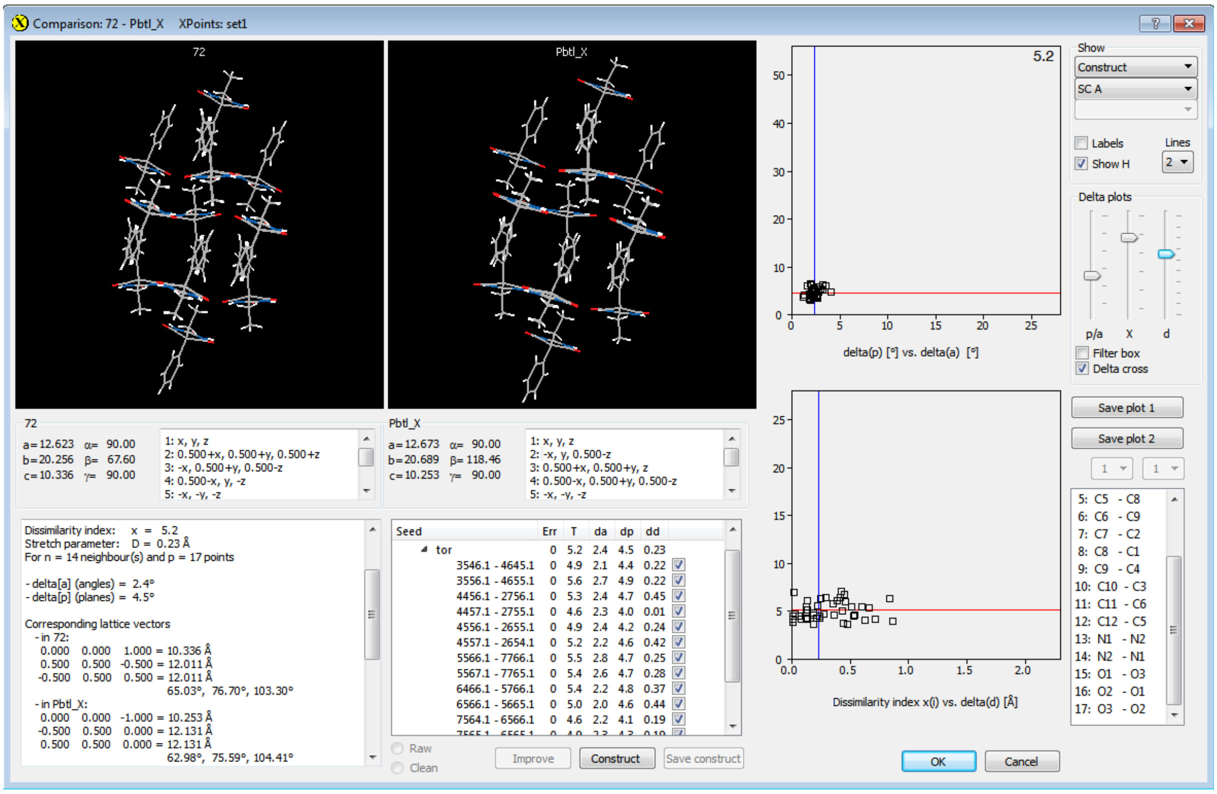

Fig. S9. Screenshot showing a summary of an *XPac* comparison [2] between the model for polymorph **X** and the theoretical candidate structure #72 reported by Day *et al.* [3] All 17 matching non-H atomic positions were used to generate geometrical parameters for the *XPac* calculations. The two structures are isostructural with an *XPac* dissimilarity index [4] of 5.2 for the complete molecule shells.

Relationship between unit cells

A transformation of #72 with the matrix $(10\bar{1}\ 010\ 001)$ leads to a *C*2/*c* structure whose lattice parameters correspond directly with those of polymorph **X**.

Predicted structure #72 [3]:

a = 12.623 Å, *b* = 20.256 Å, *c* = 10.336 Å; β = 67.60°; space group *I*2/*a*.

Transformed structure #72:

a' = 12.913 Å, *b'* = 20.256 Å, *c'* = 10.336 Å; β = 115.34°; space group *C*2/*c*.

Pbtl polymorph **X**:

a = 12.673 Å, *b* = 20.689 Å, *c* = 10.253 Å; β = 118.46°; space group *C*2/*c*.

8. Structure model for polymorph X

The following structure model for polymorph X of Pbtl was derived from that of the co-crystal of Pbtl with pentobarbital (CSD refcode LATMEA).

```
_symmetry_cell_setting monoclinic
_symmetry_space_group_name_H-M 'C 2/c'
_symmetry_Int_Tables_number 15
loop_
_symmetry_equiv_pos_site_id
_symmetry_equiv_pos_as_xyz
1 x,y,z
2 -x,y,1/2-z
3 1/2+x,1/2+y,z
4 1/2-x,1/2+y,1/2-z
5 -x,-y,-z
6 x,-y,-1/2+z
7 -1/2-x,-1/2-y,-z
8 -1/2+x,-1/2-y,-1/2+z
_cell_length_a 12.6733(11)
_cell_length_b 20.6887(19)
_cell_length_c 10.2529(7)
_cell_angle_alpha 90
_cell_angle_beta 118.458(9)
_cell_angle_gamma 90
_cell_formula_units_Z 4
loop_
_atom_site_label
_atom_site_type_symbol
_atom_site_fract_x
_atom_site_fract_y
_atom_site_fract_z
O1 O 1.0648(2) 0.42427(17) 0.6420(2)
O2 O 0.8417(2) 0.43034(18) 0.1480(2)
O3 O 0.6670(2) 0.3995(2) 0.4608(3)
N1 N 0.8648(2) 0.4116(2) 0.5488(3)
H1 H 0.87360 0.41130 0.63930
N2 N 0.9512(2) 0.42189(17) 0.3940(3)
H2 H 1.01660 0.42490 0.38470
C1 C 0.9659(3) 0.4193(2) 0.5362(3)
C2 C 0.8447(3) 0.4202(2) 0.2672(3)
C3 C 0.7329(3) 0.4021(3) 0.2777(4)
C4 C 0.7499(3) 0.4042(3) 0.4342(4)
C5 C 0.6308(3) 0.4473(3) 0.1817(4)
H3 H 0.62250 0.44810 0.08070
H4 H 0.55520 0.42980 0.17380
C6 C 0.6474(4) 0.5159(4) 0.2401(7)
H5 H 0.64130 0.51660 0.33170
H6 H 0.58520 0.54360 0.16580
H7 H 0.72670 0.53170 0.26040
C7 C 0.7229(12) 0.3296(5) 0.2317(18)
C8 C 0.7952(11) 0.2811(7) 0.3256(15)
H8 H 0.85400 0.29140 0.42410
C9 C 0.7814(13) 0.2177(6) 0.2755(18)
H9 H 0.83080 0.18460 0.33980
C10 C 0.6954(14) 0.2027(5) 0.1314(19)
H10 H 0.68600 0.15940 0.09720
C11 C 0.6231(13) 0.2512(8) 0.0375(14)
H11 H 0.56430 0.24100 -0.06100
C12 C 0.6369(12) 0.3147(7) 0.0876(17)
H12 H 0.58750 0.34780 0.02330
```

9. References

1. Zencirci N, Gelbrich T, Apperley DC, Harris RK, Kahlenberg V, Griesser UJ: **Structural features, phase relationships and transformation behavior of the polymorphs I–VI of phenobarbital.** *Cryst Growth Des* 2010, **10:**302-313.
2. Gelbrich T, Hursthouse MB: **A versatile procedure for the identification, description and quantification of structural similarity in molecular crystals.** *CrystEngComm* 2005, **7:**324-336.
3. Day GM, Motherwell WDS, Jones W: **A strategy for predicting the crystal structures of flexible molecules: the polymorphism of phenobarbital.** *Phys Chem Chem Phys* 2007, **9:**1693-1704.
4. Gelbrich T, Threlfall TL, Hursthouse MB: ***XPac* dissimilarity parameters as quantitative descriptors of isostructurality: the case of fourteen 4,5'-substituted benzenesulfonamido-2-pyridines obtained by substituent interchange involving Cf$_3$/I/Br/Cl/F/Me/H** *CrystEngComm* 2012, **14:**5454-5464.
